# *Pseudomonas putida* CSV86: A Candidate Genome for Genetic Bioaugmentation

**DOI:** 10.1371/journal.pone.0084000

**Published:** 2014-01-24

**Authors:** Vasundhara Paliwal, Sajan C. Raju, Arnab Modak, Prashant S. Phale, Hemant J. Purohit

**Affiliations:** 1 Environmental Genomics Division, CSIR-National Environmental Engineering Research Institute, Nagpur, India; 2 MEM-Group, Department of Biosciences, University of Helsinki, Helsinki, Finland; 3 Department of Biosciences and Bioengineering, Indian Institute of Technology-Bombay, Powai, Mumbai, India; Louisiana State University and A & M College, United States of America

## Abstract

*Pseudomonas putida* CSV86, a plasmid-free strain possessing capability to transfer the naphthalene degradation property, has been explored for its metabolic diversity through genome sequencing. The analysis of draft genome sequence of CSV86 (6.4 Mb) revealed the presence of genes involved in the degradation of naphthalene, salicylate, benzoate, benzylalcohol, *p*-hydroxybenzoate, phenylacetate and *p*-hydroxyphenylacetate on the chromosome thus ensuring the stability of the catabolic potential. Moreover, genes involved in the metabolism of phenylpropanoid and homogentisate, as well as heavy metal resistance, were additionally identified. Ability to grow on vanillin, veratraldehyde and ferulic acid, detection of inducible homogentisate dioxygenase and growth on aromatic compounds in the presence of heavy metals like copper, cadmium, cobalt and arsenic confirm *in silico* observations reflecting the metabolic versatility. *In silico* analysis revealed the arrangement of genes in the order: tRNA^Gly^, integrase followed by *nah* operon, supporting earlier hypothesis of existence of a genomic island (GI) for naphthalene degradation. Deciphering the genomic architecture of CSV86 for aromatic degradation pathways and identification of elements responsible for horizontal gene transfer (HGT) suggests that genetic bioaugmentation strategies could be planned using CSV86 for effective bioremediation.

## Introduction

*Pseudomonas* exhibits diverse metabolic capacities; which allow it to survive in different ecological niches, including sites contaminated with pollutants such as aromatic compounds. The required metabolic attributes are reflected by its large size genome (generally >6 Mb). Pseudomonads have been reported for their ability to exchange genetic information through horizontal gene transfer (HGT) *via* phages, plasmids, transposons and genomic islands (GIs), thus aiding in dissemination as well as evolution of new diversified metabolic pathways [1]. These processes allow sustained survival of genetic resources. Of these mobile genetic elements (MGEs), GIs have especially been reported to code for genes which render metabolic versatility, pathogenicity and heavy metal resistance to microbes [2], [3]. These capacities could be exploited through genetic bioaugmentation for *in situ* breeding of native population which not only ensures the survival of novel genetic determinants, but also helps in enhancing the bioremediation process.

*Pseudomonas putida* CSV86 (hereafter referred to as CSV86), a soil isolate, utilizes aromatic compounds like naphthalene, 1- and 2-methylnaphthalene, phenylacetic acid (PA) and *p*-hydroxyphenylacetic acid (4-HPA), salicylate, benzylalcohol, benzoate and *p*-hydroxybenzoate as the carbon source [4], [5], [6], [7]. Strain CSV86 showed a novel property of preferential utilization of aromatic compounds over glucose and co-metabolism of aromatics and organic acids [8], [9], [10], [11]. Though CSV86 lacks plasmid, the naphthalene degradation property could be transferred by conjugation which was found to be integrated in to the chromosome of the transconjugants [12].

In the present study, the reported pathways for the utilization of aromatic compounds have been annotated using the draft genome sequence of CSV86 (6.4 Mb) [13]. Genome analysis revealed additional catabolic pathways for aromatic compounds as well as heavy metal resistance. These observations were further validated by phenotypic (cell-growth and enzyme activity) experiments. Based on these analyses, bioremediation and bio-augmentation strategies can be developed for the effective remediation of ecosystems polluted with aromatic compounds.

## Materials and Methods

### CSV86 draft genome assembly, ordering and annotation

The genome of *Pseudomonas putida* CSV86 was sequenced using Roche 454 GS (FLX Titanium) platform. The 867,565 high quality reads were assembled into 228 contigs with Newbler Ver2.0, 454 assembly tool with sequence coverage of 61.08 fold and average read length of 428 bp. Ordering of contigs was performed using a tool, Mauve Contig Mover (MCM) [14] available in Mauve software (http://gel.ahabs.wisc.edu/mauve.) using *P. putida* S16 complete genome (NC_015733) as the reference. *P. putida* S16 was also used as a reference for contig scaffolding by using SIS program [15].

The genome was annotated using Rapid Annotations using Subsystems Technology (RAST) v4.0 [16] and NCBI PGAAP (Prokaryotic Genomes Automatic Annotation Pipeline) (http://www.ncbi.nlm.nih.gov/genomes/static/Pipeline.html). In NCBI PGAAP, the 228 contigs were trimmed down to 209 due to quality check and preprocessing of sequences. These contig were later processed for annotation. The annotation by both RAST and NCBI PGAAP tool was used to describe the genome of CSV86 in this paper.

This Whole Genome Shotgun project has been deposited at DDBJ/EMBL/GenBank under the accession no. AMWJ00000000. The version described in this paper is the first version, AMWJ01000000.

### Comparative genomics and phylogenetic relationship

#### Taxonomic relationship

Phylogenetic relationship of CSV86 was established using 16S rRNA gene sequences of 38 completely sequenced *Pseudomonas* species from NCBI database. The alignment was carried out using ClustalW and the phylogenetic tree was constructed using the maximum likelihood algorithm (Hasegawa-Kishino-Yano model) with MEGA 5.2 [17]. In addition, MEGA 5.2 was also used for alignment and constructing phylogenetic tree using promoter sequences of naphthalene degradation genes.

#### Sequence variation in metabolic genes

Primary DNA and protein sequences of CSV86 were compared with other closely related species for similarity in catabolic pathways using NCBI blast tools such as megaBLAST and BLASTp, respectively. Genes involved in the degradation of aromatic compounds in CSV86 were identified using RAST and NCBI PGAAP annotation along with available literature, KEGG [18] and Metacyc [19] databases.

#### Comparative genome analysis using BRIG and Mauve

BRIG (BLAST Ring Image Generator) [20] software was used for the circular representation of multiple genome comparison. The draft genome of CSV86 was used as the reference genome and was compared with genome of *P. putida* S16 (NC_015733), *P. putida* KT2440 (NC_002947), *P. entomophila* L48 (NC_008027) and *P. stutzeri* CCUG 29243 (NC_018028).

Progressive alignment function of Mauve software with default settings was used to compare the homology among naphthalene degradation pathway genes reported from various *Pseudomonas* genomes. The draft genome of CSV86 was aligned against complete genome of *P. stutzeri* CCUG 29243 (NC_018028), *Pseudomonas* sp. ND6 plasmid pND6-1 (NC_005244), *P. putida* plasmid NAH7 (NC_007926) and *P. fluorescens* strain PC20 plasmid pNAH20 (AY887963).

### Prediction of GIs and mobile genetic elements

To predict the GIs, GC-profile, a web based tool [21] was used to compute the GC content variation in DNA sequences. These islands are marked by certain features such as the presence of mobility genes, difference in the G+C content as compared to the rest of genome, codon usage, tRNA genes and direct repeats [22]. Some of these features were manually identified in the genome for the validation of GIs. Also, conserved insertion sequences (IS) elements in CSV86 genome were identified using IS Finder (http://www-is.biotoul.fr/) to further support the presence of GI [23].

### Validation of selected genotype by wet experiments

#### Growth

Strain CSV86 was grown on 150 ml minimal salt medium (MSM) [5] in 500 ml capacity baffled Erlenmeyer flasks at 30°C on a rotary shaker (200 rpm) supplemented aseptically with vanillin, veratraldehyde, ferulic acid, phenylalanine or tyrosine (0.1%). The cell growth was observed spectrophotometrically at 540 nm.

#### Preparation of cell-free extracts

CSV86 cells grown on phenylalanine (0.1%) or glucose (0.25%) till late-log phase were harvested and washed twice with Tris-malaete buffer (200 mM, pH 6.0). Cells were re-suspended in ice-cold Tris-malaete buffer (1∶4 [wt/vol]) and sonicated at 4°C with four cycles of 15 pulses each (1 s pulse, 1 s interval, cycle duration 30 s, output 15 W) using an Ultrasonic processor (GE130). The cell lysate was centrifuged at 37,000 ×*g* for 30 min. The clear supernatant obtained was referred to as the cell-free extract and used as the source of enzyme. Protein was estimated by the method of Bradford [24] using bovine serum albumin as the standard.

#### Enzyme assay

Homogentisate 1,2-dioxygenase activity was monitored by measuring the rate of O_2_ consumption at 30°C using an oxygraph (Hansatech) fitted with a Clarke's O_2_ electrode. The reaction mixture (1 ml) contained Tris-malaete buffer (200 mM, pH 6.0), homogentisate (2.5 mM) and an appropriate amount of enzyme. The enzyme activity was calculated as nmol of O_2_ consumed per min. The specific activity is reported as nmol of O_2_ consumed min^−1^ mg^−1^ protein.

#### Heavy metal resistance

CSV86 was grown on 150 ml modified minimal salt medium (MSM, medium contained Tris, 8 g; KH_2_PO_4_,0.2 g; NH_4_NO_3_, 1 g; MgSO_4_.7H_2_O, 100 mg; MnSO_4_.H_2_O, 1 mg; CuSO_4_.5H_2_O, 1 mg; FeSO_4_.7H_2_O, 5 mg; H_3_BO_3_, 1 mg; CaCl_2_.2H_2_O , 1 mg; NaMoO_4_, 1 mg; pH 7.5) in 500 ml capacity baffled Erlenmeyer flasks at 30°C on a rotary shaker (200 rpm) supplemented aseptically with naphthalene (0.1%) or glucose (0.25%) and appropriate concentration of heavy metals such as copper, cadmium or cobalt (0.5 or 1 mM) and the growth was monitored.

## Results and Discussion

### *Pseudomonas putida* CSV86 genome features and comparative genomics

**Figure 1:**
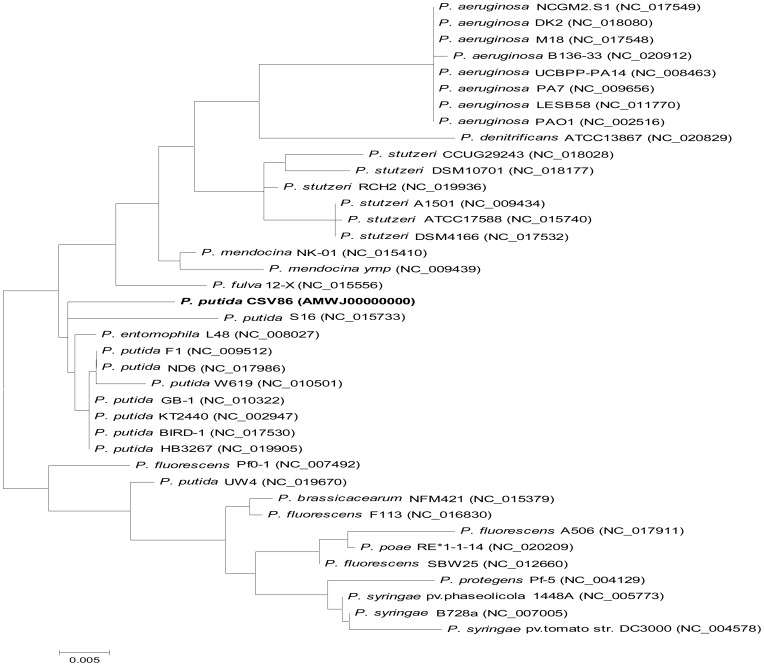
Phylogenetic neighbor-joining tree of *Pseudomonas putida* CSV86. The tree is constructed from 16S rRNA gene sequences from 38 completely sequenced *Pseudomonas* spp. The phylogenetic analysis was performed using MEGA 5.2 and the resultant Maximum Likelihood tree shows close taxonomic relationship of *P. putida* CSV86 to *P. putida* S16.

**Figure 2:**
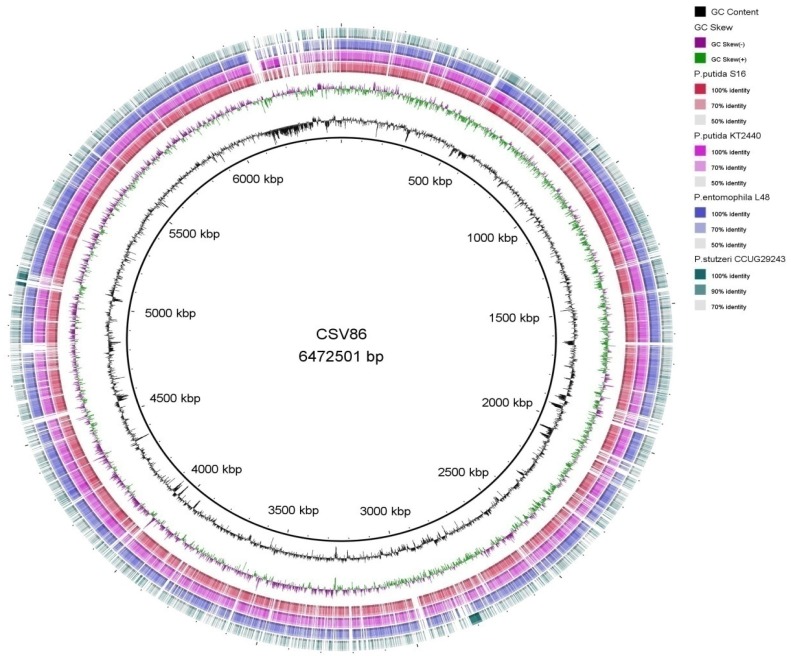
BLAST comparison of draft genome of *Pseudomonas putida*CSV86 against four *Pseudomonas* species, using BRIG. The innermost rings depict GC content (Black) and GC Skew (purple/green) followed by concentric rings of query sequences colored according to BLAST identity. The outermost rings depict genomes of the following microbes- *P. putida* S16 (Red), *P. putida* KT2440 (Pink) *P. entomophila* L48 (Blue), and *P. stutzeri* CCUG 29243 (Green).

The 6,469,780 bp draft genome of CSV86 is almost close to sequenced *Pseudomonas* genomes (Table S1); and assembled into 209 contigs that have been annotated by NCBI PGAAP into 5,836 coding sequences (CDSs) as shown in Table 1. RAST analysis divided CSV86 genome into different metabolic subsystems including catabolic pathways for various aromatic compounds (Figure S1). The phylogenetic tree of 16S rRNA gene of CSV86 showed taxonomic relationship with *Pseudomonas putida* S16 sharing 98% homology (Figure 1). This was further supported by SIS program, wherein CSV86 draft genome was assembled into 8 scaffolds (around 0.3 Mb of the genome was unmapped in the scaffold) against *P. putida* S16. The analysis of ordered draft genome of CSV86 with BRIG software showed ∼70% identity with *P. putida* S16, *P. putida* KT2440 and *P. entomophila* L48 except *P. stutzeri* CCUG 29243 (<70%) (Figure 2); with gaps observed in the region 6100–6500 kbp. Similarity search of the gapped region using BLASTn with default parameters, revealed genes coding for chromosome replication initiator protein *dnaA* and other proteins involved in replication. A gene cluster with *dnaA* gene was identified i.e. *rnpA-rpmH-dnaA-dnaN-recF-gyrB*. The *oriC* (replication origin) has been reported to be present in this intergenic region [25], [26]. Therefore, it may be postulated that *oriC* region is located in this region of CSV86 genome.

**Table 1:** Features associated with genome of *P. putida* CSV86 according to NCBI PGAAP.

FEATURE	CHROMOSOME
Length (bp)	6,469,780 bp
Number of contigs≠	209
GC content (%)	61.85
Sequencing coverage	61.08×
t-RNA genes	60
CDSs*	5,836
Hypothetical proteins	1,689

* CDSs: coding region, coding sequence.

≠ There are 228 contigs according to Newbler Ver2.0, 454 assembly tool.

### Mining of aromatic compound degradation pathways in CSV86

The industrial revolution has led to the introduction of new pollutants in the environment; which also ushered the evolution of new catabolic pathways [27]. The absence or withdrawal of such selective pressures often leads to the loss of the catabolic property, if it is plasmid mediated; and even in cases of genome organization, where it is associated with MGEs such as GIs. These features play a significant role in the evolution of community where these evolved microbes can be ideal candidates for effective bioremediation either alone or in consortium [28].

**Figure 3:**
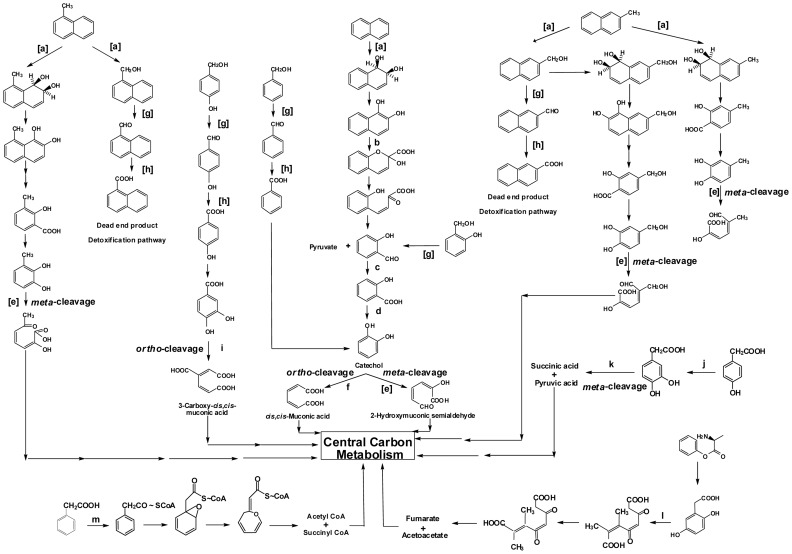
The metabolic pathways for aromatic compounds in *Pseudomonas putida*CSV86. Enzymes involved are: a, naphthalene dioxygenase; b, 1,2-dihydroxynaphthalene dioxygenase; c, salicylaldehyde dehydrogenase; d, salicylate hydroxylase; e, catechol 2,3-dioxygenase; f, catechol 1,2-dioxygenase; g, benzyl alcohol dehydrogenase; h, benzaldehyde dehydrogenase; i, 3,4-dihydroxybenzoate-3,4-dioxygenase; j, 4-hydroxyphenylacetic acid hydroxylase; k, 3,4-dihydroxyphenylacetic acid dioxygenase; l, homogentisate 1,2-dioxygenase; m, phenylacetyl-CoA ligase. Enzymes with wide-substrate specificity involved in various pathways in CSV86 are indicated in square bracket (4, 5, 6, 7).

**Figure 4:**
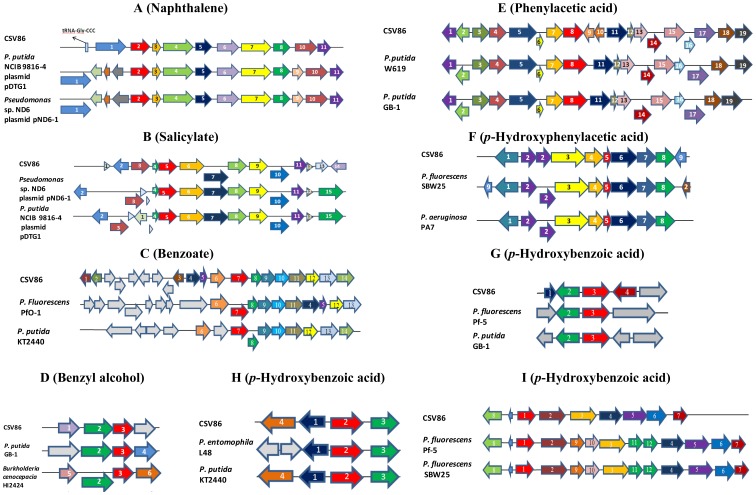
Gene organization of aromatic degradation pathways reported to be functionally characterized from *Pseudomonas putida* CSV86. **A**. Naphthalene pathway (Contig 105), **B**. Salicylate pathway (Contig 69), **C**. Benzoate pathway (Contig 175), **D**. Aromatic alcohol degradation pathway (Contig 119), **E**. Phenylacetic acid pathway (Contig 88), **F**, Hydroxylphenylacetic acid pathway(Contig 7) and **G–I**, 4-Hydroxybenzoate (Contig 107, 99, 118). For details refer to Figures S2, S3, S6, S7, S8, S9, S10.

**Figure 5:**
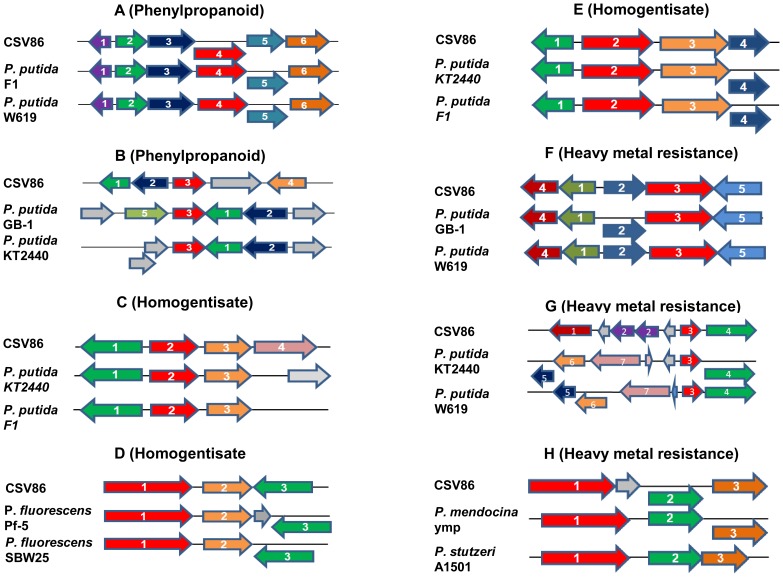
New aromatics degradation pathways genes identified in *Pseudomonas putida* CSV86 by genome analysis. **A–B**. Phenylpropanoid pathway genes (Contig 115, 220), **C–E**. Homogentisate pathway genes (Contig 27, 99, 177), **F–H**. Copper resistance genes (Contig 19). For details refer to Figures S11, S12, S12.

The *in-silico* analysis of the CSV86 genome revealed the genes coding for enzymes involved in the metabolic pathways which are biochemically characterized earlier from CSV86 (Figure 3) and their arrangement on the genome (Figure 4) as well as newly identified pathways for the catabolism of aromatic compounds (Figure 5).

#### Naphthalene degradation pathway

CSV86 can utilize naphthalene and its derivates such as 1- and 2-methylnaphthalenes as the sole source of carbon and energy *via* ring-hydroxylation pathway (Figure 3), while side-chain hydroxylation pathway leads to its detoxification [4], [5]. In CSV86, naphthalene catabolic pathway is initiated by naphthalene 1,2-dioxygenase (a three-component system) which catalyzes the hydroxylation of the aromatic ring to yield 1,2-dihydroxynaphthalene as a upper pathway (contig 105). This diol is further sequentially oxidized to catechol *via* lower pathway (contig 69), which enters the tricarboxylic acid cycle (TCA) after *meta* ring-cleavage (Figure 3) [4]. Using BLASTp, amino acid sequences of CSV86 naphthalene degrading upper and lower pathway genes (Table S2) were compared with that of other reported bacteria. It was observed that upper pathway amino acid sequences shares higher homology and hence are more conserved than that in lower pathway (Table S3).

Both the *nah* and *sal* operon of CSV86 showed similarity with *Pseudomonas putida* NCIB 9816-4 plasmid pDTG1 and *Pseudomonas* sp. ND6 plasmid pND6-1 (Figure 4A & 4B; Figures S2 & S3). Interestingly, in contig 105, the arrangement of genes observed was tRNA^Gly^, integrase followed by *nah* operon in the order *nahAa,Ab,Ac,Ad, BFCED*. This arrangement is a characteristic feature of a GI for e.g. *clc* element [29]. The *sal* operon consists of 9 genes organized as *nahGTHILMOKJ* with the regulatory gene *nahR* present downstream of *nahG* gene. The regulation of *nah* genes is controlled by *nahR*, which is in turn induced by salicylate [30], [31]. In CSV86 a transposase encoding gene is present upstream of *nahR* gene which is missing in the plasmids being compared (Figure 4B & Figure S3).

#### Regulation of nah operon

The genome sequence analysis of CSV86 revealed that the naphthalene pathway is under the control of LysR family of transcription regulators (LTTRs) [32], [33]. We have analyzed the differential regulation of naphthalene degradation pathway in CSV86 with *P. stutzeri* CCUG 29243 (NC_018028) (chromosomally coded) and *Pseudomonas putida* plasmid NAH7 DNA, strain G7 (AB237655). NahR protein is essential for the activation of both the upper and lower operon of naphthalene pathway in the presence of salicylate [34]. The binding site for NahR with the promoter for *Pnah* and *Psal* are reported to be located at 60 bp upstream to transcriptional start site [34], [35]. Therefore, the promoter data for *nahAa* (upper pathway), *nahG* (lower pathway) and also the coding sequences for NahR protein was analyzed. The consensus binding sequences of NAH7 promoter has two *cis*-acting elements situated 6 bp apart that interact with NahR protein [36]. The *nahAa* promoter of CSV86 and *P. stutzeri* are identical with the reported NAH7 binding site for NahR protein. Both these promoters have an additional *cis*-acting element with one base pair substitution and 4 bp spacing between the *cis*-acting elements. Whereas, *nahG* promoter has a base pair substitution (TGAT is changed to TAGT) in both the chromosomal promoters, with 4 bp separating the two *cis*-acting elements (Table 2). A phylogenetic tree of *nahR, nahAa* and *nahG* promoter sequences was also constructed. In all the three promoter sequence comparisons, CSV86 and *P. stutzeri* were grouped in same cluster (Figure S4).

**Table 2:** Consensus sequence of nahR binding site in *nahAa* and *nahG* gene obtained from *P. putida* plasmid NAH7.

Promoter	NahR binding site
**pNah7**	-CGCAnT**AT**TCAyGyTGuTG**A**T nnAnnAnnTnnn-
**PnahAa**	
CSV86	-GACAT T**AT**TCATATTAGTG**A**T ACTAA T**AT**TCA TTTATGGT TTATTGAC-
*P. stutzeri*	-GACAT T**AT**TCATATTAGTG**A**T ACTAA T**AT**TCATTTATGGT TTATTGAC-
**PnahG**	
CSV86	-TAGTG T**AT**TTATCAATAGT TATGGCTTCGCTACTGTT-
*P. stutzeri*	-TAGTG T**AT**TTATCAATAGT TATGGCTTCGCTACTGTT-

Table shows consensus sequence of NahR binding site form *P. putida* plasmid NAH7 as reported by Schell *et al.*, 1989. The sequences used in Figure S4, shown the homologous *cis*-acting NahR regulated elements of *nahAa* and *nahG* genes (*nah* and *sal* operons, respectively) in case of *P. putida* CSV86 and *P. stutzeri* CCUG 29243 genomes. The bold type face alphabets indicate nucleotides required for NahR activation of NahR-regulated promoters. (n: no nucleotide preference, Y: pyrimidine; U: purine).

The NahR of CSV86 interestingly showed 100% identity with protein from *P. stutzeri* (chromosomally located) as compared to 81% identity with NAH7 (plasmid encoded) protein. The substitution of methionine to isoleucine in NahR protein of NAH7 altered the specificity of protein to salicylate and allowing salicylate analog like benzoate to act as an inducer [37]. In CSV86 and *P. stutzeri*, NahR protein at 116^th^ position has isoleucine (Figure S5). However in CSV86, the enzymes responsible for naphthalene and salicylate degradation are inducible in nature. Benzoate does not induce these operons as the benzoate grown cells failed to respire on naphthalene or salicylate and showed no activity of enzymes involved in naphthalene or salicylate degradation [5].

#### Benzoate degradation pathway

In CSV86 benzoate degradation is initiated with the incorporation of molecular oxygen by benzoate dioxygenase (encoded by *benABC* genes, a two-component system) to yield catechol which enters the central carbon metabolism *via* β-ketoadipate pathway after *ortho*-cleavage (Figure 3) [5], [38]. The details of the genes for benzoate degradation that are present in contigs 175, 103, 118 and 116 are described in Table S2. The CSV86 genome has the presence of complete benzoate utilization system including the regulatory option of the *benABC* operon i.e., transcriptional activator BenR with benzoate as effector; and utilization of catechol regulated *via* CatR with *cis,cis*-muconate as effector [38]. In *P. fluorescnes* Pf0-1 the *catBC* and *catR* genes are located between genes encoding benzoate MFS transporter and catechol 1,2-dioxygenase while, in CSV86 these genes are located upstream to the benzoate cluster. The *catBC* genes are absent in this cluster of *P. putida* KT2440 (Figure 4C, Figure S6).

The optional genes for benzoate degradation were also identified in CSV86 genome, which encode *p*-hydroxybenzoyl-CoA thioesterase (catalyses the conversion of *p*-hydroxybenzoyl-CoA to *p*-hydroxybenzoate) and 5-carboxymethyl-2-hydroxymuconate Delta-isomerase (catalyses the conversion of 5-carboxymethyl-2-hydroxymuconate to form 5-carboxy-2-oxohept-3-enedioate) located in contig 60 and 103, respectively. Besides salicylate hydroxylase in contig 69 (*sal* operon), contig 103 also showed the presence of an additional salicylate hydroxylase with 23% identity.

#### Aromatic alcohol degradation pathway

Although the detoxification pathway of methylnaphthalenes closely resembles to the side-chain hydroxylation of toluene degradation, CSV86 failed to utilize toluene or xylene as the sole source of carbon and energy. Interestingly, strain could grow on benzyl alcohol, 2- and 4-hydroxy benzyl alcohol (Figure 3) [5]. The key enzymes of the aromatic alcohol metabolism, benzyl alcohol dehydrogenase (BADH) and benzaldehyde dehydrogenase (BZDH), have been purified and were found to be wide-substrate specific and shown to catalyze the conversion of 1- and 2-hydroxymethylnaphthalene to respective naphthoic acids (dead end products) [5], [6].

In CSV86, the gene cluster encoding BZDH and BADH was located in contig 119. The proposed BZDH or NAD^+^-dependent aryl aldehyde dehydrogenase gene was located adjacent to BADH, aryl alcohol dehydrogenase gene in CSV86 and shares homology with *Pseudomonas putida* DOT-T1E (aldehyde dehydrogenase, 87%). In CSV86 gene encoding for transcriptional regulator (AraC family) was located downstream to the gene encoding putative benzaldehyde dehydrogenase oxidoreductase protein, which is absent in the same cluster of *Pseudomonas putida* GB-1 and *Burkholderia* sp. (Figure 4D, Figure S7, Table S2).

#### Phenylacetic and p- hydroxyphenylacetic acid degradation pathway

CSV86 metabolizes phenylacetic acid (PA) and *p*-hydroxyphenylacetic acids (HPA) using an unconventional ‘aerobic hybrid’ pathway (Figure 3). PA is first activated to its CoA-thioester (phenylacetyl CoA) by phenylacetyl-CoA ligase, which through a series of CoA-thioester intermediates ultimately leads to the formation of succinyl-CoA and acetyl-CoA that enters the central metabolic pathway (Figure 3) [7], [39]. In CSV86 the PA catabolic genes (contig 88, Table S2) were organized in the order as observed in *Pseudomonas putida* W619 and *P. putida* GB-1 (Figure 4E, Figure S8).

4-HPA degradation pathway in CSV86 follows homoprotocatechuate route which involves initial hydroxylation of 4-HPA followed by ring cleavage (Figure 3) [7]. The pathway genes of 4-HPA in CSV86 were present in contig 7, 120, 175 and 216 (Table S2). There were two copies of each *hpaD* (contig 7 and 120) and homoprotocatechuate degradative operon repressor gene (contig 7 and 216). The genes present in CSV86 contig 7 were organized as *hpaAGEDFHI* while, in *Pseudomonas fluorescens* SBW25 homoprotocatechuate degradative operon repressor gene was present upstream of this *hpa* cluster (Figure 4F, Figure S9).

#### p-Hydroxybenzoate degradation pathway

In CSV86, *p*-hydroxybenzoate degradation is initiated by ring-hydroxylating 4-hydroxybenzoate 3-monooxygenase (PobA) to yield 3,4-dihydroxybenzoate (protocatechuate) which is further metabolized by *ortho* ring-cleavage to yield carboxy-*cis,cis*-muconate by protocatechuate dioxygenase (PcaGH, Figure 3). The later part of the pathway was catalyzed by the *pcaBCDIJF* gene products which are also involved in the degradation of benzoate.

The genes for 4-hydroxybenzoate pathway were distributed in contig 99 (*pcaHG*), 107 (*pobA*), 118 (*pcaRKFTBDC*) and 175 (*pcaIJ*, Table S2) in CSV86. Like *Pseudomonas fluorescens* Pf-5 and *P. putida* GB-1, *pobA* gene in CSV86 (contig 107) was located downstream to the gene encoding transcriptional regulator PobR (AraC family) (Figure 4G, Figure S10A). The *pcaHG* genes were located in contig 99 and present downstream of zinc metalloprotease superfamily, as observed in *P. entomophila* L48 and *P. putida* KT2440 (Figure 4H, Figure S10B). A transporter, PcaK, is involved in the transport of 4-hydroxybenzoate across the membrane and reported to be located in between *pcaR* and *pcaF* in *P. putida*, as can also be seen in contig 118 in CSV86 (Figure 4I, Figure S10C). The expression of *pcaK* gene has been shown to be repressed by benzoate, suggesting cells prefer benzoate instead of 4-HPA when given together [40]. The *pca* genes have been shown to be arranged in a single cluster in *P. fluorescens* [41], while in CSV86 they were segregated in different contigs (Figure 4, Table S2, Figure S10).

### Identification of additional aromatic compound degradation pathways

#### Phenylpropanoid degradation pathway

Although lignin degradation pathways are shown in fungi by enzymes such as lignin peroxidases, manganese peroxidases, laccases etc [42]; aromatic degrading bacterial species are also reported to metabolize lignin [43]. Vanillin, ferulic acid, veratraldehyde, coniferyl aldehyde, β-coniferylether are identified as degradation intermediates of lignin. In *Pseudomonas* sp. strain HR199, the feruloyl-CoA synthetase (encoded by *fcs*) activates ferulic acid to its CoA-thioester followed by hydration and non-oxidative cleavage by enoyl-CoA hydratase/aldolase (encoded by *ech*) to form vanillin and acetyl Co-A [44]. Vanillin is further converted to vanillate and later protocatechuate by vanillin dehydrogenase (encoded by *vdh*) and vanillin monooxygenase (encoded by *vanAB*) or vanillate-O-demethylase (encoded by *vanA* and *vanB*), respectively [41], [44]. In CSV86, genes involved in the phenylpropanoid degradation were segregated in different contigs (Figure 5A&B, Table S2, Figure S11). The *fcs-vdh-ech* genes formed a cluster in contig 115 (Figure 5A, Figure S11A), *vanAB* genes in contig 119; and Vanillate O-demethylase oxygenase subunit, *vanA* and vanillate O-demethylase oxidoreductase, *vanB* along with the transcriptional regulator for ferulate or vanillate catabolism in contig 220 (Figure 5B, Figure S11B).

Strain CSV86 showed good growth on MSM supplemented with vanillin, veratraldehyde, or ferulic acid (0.1%). These observations suggest that the lignin degradation intermediates can be used as the sole source of carbon and energy and reflects the existence of functional phenylpropanoid metabolic pathway in CSV86. However, cells failed to utilize lignin (lignin sulphonic acid) as the sole source of carbon and energy (data not shown).

#### Homogentisate degradation pathway

Homogentisate is a metabolic intermediate of aromatic amino acid pathways. Phenylalanine *via* tyrosine is converted to 4-hydroxyphenylpyruvate (by PhhABC). The generated 4-hydroxyphenylpyruvate, is then transformed to homogentisate (2,5-dihydroxyphenylacetic acid) by 4-hydroxyphenylpyruvate dioxygenase (encoded by *hpd* gene) [41]. Homogentisate 1,2-dioxygenase (HmgA) is the first enzyme of the homogentisate pathway which catalyses the transformation of homogentisate to 4-maleylacetoacetate. Isomerization of 4-maleylacetoacetate by maleylacetoacetate isomerase (HmgC) leads to the formation of fumarylacetoacetate, which is finally hydrolyzed by fumarylacetoacetase (HmgB) generating fumarate and acetoacetate [45], [46]. The *hpd* gene is present along with *hmg* genes in *Pseudomonas syringae, Pseudomonas stutzeri* and *Pseudomonas mendocina*, whereas in *P. putida* these are scattered; in *P. aeruginosa* the *hpd* gene is clustered with *phh* genes [41]. In CSV86, *phh*, *hpd* and *hmg* genes were segregated in different clusters (Figure 5C, D & E; Figure S12,Table S2). The *phh* genes were present in the contig 177 with the arrangement of *phhR* (phenylalanine hydroxylase transcriptional activator) and *phhABC* genes similar to *P. putida* KT2440 and *P. putida* F1 (Figure 5C, Figure S12C). The *hpd* gene was present in contig 99 (Figure 5D, Figure S12B) and 134. The gene coding for transcriptional regulator, TetR family, was present upstream to *hpd* gene in CSV86 (contig 99), *P. fluorescens* Pf-5 and SBW25. The clustering of *hmg* genes (contig 127) was similar to *P. putida* F1 and KT2440, with the gene coding for transcriptional regulator (IclR family) being transcribed in reverse direction to *hmgABC* genes (Figure 5E, Figure S12A).

Strain CSV86 showed good growth on MSM supplemented with phenylalanine or tyrosine (0.1%) as the sole source of carbon and energy. Cell-free extract prepared from the cells grown on phenylalanine showed the activity of homogentisate dioxygenase (specific activity 49.9 nmol min^−1^ mg^−1^protein) while glucose grown cells failed to do so. These results suggest that the homogentisate pathway is functional in CSV86 and the enzyme is inducible in nature (data not shown).

### Identification of heavy metal resistance genes in CSV86

Bioremediation of soils co-contaminated with heavy metals and organics pose a major environmental challenge. Therefore, bacteria harboring the properties of heavy metal resistance as well as aromatic compound degradation would be highly beneficial. The metal resistance is achieved by employing efflux pumps or enzymatic detoxification or bioaccumulation (intracellular/surface sequestration) or in combinations. Genes involved in heavy metal resistance have been found to be encoded by plasmids [47], [48] or chromosome [49]. Chromosomal coded efflux system for cadmium resistance has been reported in *Bacillus* as well as for arsenic and antimony resistance in *E. coli* [50]. *Cyanobacterium synechocystis* PCC6808 was also found to contain a homolog to Czc (cadmium, zinc, and cobalt resistance system) and genes apparently involved in arsenic and copper transport [51].

The genome of CSV86 was found to harbor heavy metal resistance genes for copper (Figure 5F–H), cadmium, cobalt and arsenic (Table S4). The copper resistance genes were dispersed in CSV86 genome (contig 19, 153, 82) with a majority being in contig 19 (Figure 5F, Figure S13A). The genes encoding copper sensory histidine kinase (*cusS*), copper-sensing two-component system response regulator (*cusR*) and copper tolerance were also found to be located in the same contig (Figure 5G and H; Figure S13B and C). Like copper, arsenic resistance genes were also located in contig 153, with the exception of arsenic reductase gene in contig 82. Genes for cobalt, chromate, cadmium, zinc and lead resistance were also mapped during the genome analysis.

Strain CSV86 showed good growth on glucose or naphthalene in the presence of heavy metals like copper, cadmium or cobalt at 0.5 as well as at 1 mM concentration, suggesting the ability of strain to express the tolerance/resistance to these heavy metals (data not shown).

### Genetic bioaugmentation

Bioaugmentation using genetically engineered microorganisms or consortia has been reported as an alternative strategy to enhance the bioremediation of contaminated sites [52], [53], [54]; however the bio-safety issues pose a concern with these modified bacteria. A wild isolate with its ability to transfer catabolic genes through natural processes (HGT) such as conjugation, may provide a better solution to contain and remediate these compounds [55], [56]. *Pseudomonas putida* IncP-9 TOL plasmid pWW0, has been studied for genetic bioaugmentation of soil, wastewater and aerobic microbial granules [57]. In another example, dissemination of plasmid pGKT2 harboring catabolic genes for hexahydro-1,3,5-trinitro-1,3,5,-triazine (RDX) degradation was studied by means of conjugation between the *Gordonia* sp. KTR9 and the native population of the contaminated site, so as to enhance the efficiency of bioremediation [58]. Genetic bioaugmentation *via* self-transmissible catabolic genes by donor bacteria have stability issues in host bacteria. The other options for HGT events are mediated by MGEs such as plasmids, GIs, transposons, integrons and phages [55], [56]. MGEs have been shown to play significant role in supporting various types of genomic rearrangement. HGT through GIs provides a better and desirable advantage over plasmids as these elements are integrated in host chromosome resulting in a stable genotype [3]. Therefore, selective pressure for the survival of genotype is not essential for better bioremediation capability and efficiency. These shuffling introduce new gene clusters in a recipient bacteria guided through stressed conditions of environment. IS elements are also associated with transfer of metabolic loci and are therefore evolutionarily significant in bacterial genomes. They are generally less than 2.5 kb in length and encode a protein that is involved in transposition [59], [60]. Using IS Finder, we report existence of these elements in CSV86 genome (Table S5); however none of these are present in vicinity to degradation pathway genes which are associated with integrase (Figure 4A, Figure S2).

Attempts to isolate plasmid from CSV86 were unsuccessful. Further, Southern hybridization suggests that naphthalene degradation genes were localized in the genome. The naphthalene degradation property of CSV86 can be transferred by conjugation to *Stenotrophomonas maltophila* CSV89 with the transconjugants thus obtained preferentially metabolizing aromatic compounds over glucose. However, these properties were found to be unstable when transconjugants were grown on rich medium [12]. These results indicate probable involvement of GI in naphthalene degradation capability of CSV86. Comparative analysis of CSV86 genome with genome of established naphthalene degrading strains like *P. stutzeri* CCUG 29243, *Pseudomonas* sp. ND6 plasmid pND6-1, *P. putida* plasmid NAH7 and *Pseudomonas fluorescens* strain PC20 plasmid pNAH20 was performed. Analysis revealed that naphthalene and salicylate degrading gene clusters of CSV86 and *P. stutzeri* CCUG 29243 shares a high degree of homology at nucleotide sequence and showed the presence of genes encoding integrase (Figure S14) and transposase (Figure S15) upstream to both (*nah* and *sal*) operons. This feature was found to be absent for naphthalene degrading genes encoded by plasmids (pND6-1, NAH7 and pNAH20). This observation suggests the presence of GI or conjugative element(s). GIs have specific integration site (near or in tRNA gene) and lower G+C content compared to rest of the genome. The GC-profile tool, which calculates the compositional heterogeneity of DNA sequences, also postulates the presence of GI in CSV86 genome. The analysis of contig 105 which contained genes encoding for naphthalene upper pathway revealed that there was a marked difference in G+C content between the region containing naphthalene upper pathway genes (90555–100230) with the rest of the contig DNA (1–90554) suggesting possible insertion of GI in this region (Figure S16). This is supported by the occurrence of genes encoding for tRNA-Gly and integrase, located just upstream to the upper pathway genes of naphthalene degradation. Neither tRNA-Gly nor difference in the G+C content was observed in the contig 69 which encodes salicylate pathway.

## Conclusions

The analysis of draft genome of *Pseudomonas putida* CSV86, which encodes for 5836 ORFs revealed the presence of complete catabolic pathway for naphthalene degradation with more than 95% homology with reported coding sequences for *P. stutzeri* CCUG 29243. Identification of the GI at tRNA^Gly^ containing naphthalene degradation genes supports the ability to transfer the property by conjugation and the stability of this property. The identification of additional degradative and metal resistance genotype supported by phenotypic experiments further displays the diversity of CSV86. The degradative capacities associated with conjugation capabilities make this bacterium a possible donor in safe dissemination of catabolic potential for the process of genetic bioaugmentation.

## Supporting Information

Figure S1 Subsystem distribution of *Pseudomonas putida* CSV86 genome in RAST.
(TIF)

Figure S2 Organisation and comparison of catabolic genes involved in naphthalene degradation (*nah* operon) in *P. putida* CSV86 (contig 105) against *P. putida* NCIB 9816-4 plasmid pDTG1 (NC_004999), *Pseudomonas* sp. ND6 plasmid pND6-1 (NC_005244), *Acidovorax* sp. JS42 (NC_008782) and *Leptothrix cholodni* SP-6 (NC_010524). Analysis was performed using RAST.
(TIF)

Figure S3 Organisation and comparison of catabolic genes involved in salicylate acid degradation (*sal* operon) in *P. putida* CSV86 (contig 69) against *Pseudomonas* sp. ND6 plasmid pND6-1 (NC_005244), *P. putida* NCIB 9816-4 plasmid pDTG1 (NC_004999), *P. putida* MT53 plasmid pWW53 (NC_008275) and *Dechloromonas aromatica* RCB (NC_007298). Analysis was performed using RAST.
(TIF)

Figure S4 Phylogenetic tree showing comparison of *nahR* (4A), *nahAa* (4B) and *nahG* (4C) promoter sequences of *P. putida* CSV86 (AMWJ00000000), *P.stutzeri* CCUG 29243 (NC_018028) and *P. putida* plasmid NAH7 (NC_007926).
(TIF)

Figure S5 Alignment of NahR amino acid sequence of *P. putida* CSV86 (NZ_AMWJ01000062.1) with that of *P. putida* plasmid NAH7 (NC_007926) and *P.stutzeri* CCUG 29243 (NC_018028). The pink highlighted bar indicates residue 116: Methionine in plasmid NAH7 and Isoleucine in both CSV86 and *P. stutzeri*. Similarly, the blue highlighted bar indicates residue 248 (Arginine in all the three); Green bar indicates residue 132 (Arginine in all the three) and Grey bar indicates residue 169 (Asparagine in all the three).
(TIF)

Figure S6 Organisation and comparison of catabolic genes involved in the benzoate pathway in *P. putida* CSV86 (contig 175) against *P. fluorescens* PfO-1 (NC_007492), *P. putida* KT2440 (NC_002947), *P. putida* F1 (NC_009512) and *P. putida* W619 (NC_010501). Analysis was performed using RAST.
(TIF)

Figure S7 Organisation and comparison of benzyl alcohol dehydrogenase and benzaldehyde dehydrogenase genes in *P. putida* CSV86 (contig 119) against *P. putida* GB-1 (NC_010322), *Burkholderia cenocepacia* HI2424 (NC_008544), *Burkholderia cenocepacia* AU 1054 (NC_008060) and *Burkholderia ambifaria* AMMD (NC_008392). Analysis was performed using RAST.
(TIF)

Figure S8 Organisation and comparison of catabolic genes involved in phenylacetic acid degradation in *P. putida* CSV86 (contig 88) against *P. putida* W619 (NC_010501), *P. putida* GB-1 (NC_010322), *P. putida* KT2440 (NC_002947) and *P. putida* F1 (NC_009512). Analysis was performed using RAST.
(TIF)

Figure S9 Organisation and comparison of catabolic genes involved in 4-hydroxy phenylacetic acid degradation in *P. putida* CSV86 (contig 7) against *P. fluorescens* SBW25 (NC_012660), *P. aeruginosa* PA7 (NC_009656), *P. aeruginosa* UCBPP-PA14 (NC_008463) and *P. aeruginosa* PAO1 (NC_002516). Analysis was performed using RAST.
(TIF)

Figure S10 Organisation and comparison of catabolic genes involved in *p*hydroxy benzoate degradation in *P. putida* CSV86 against *P. fluorescens* Pf-5 (NC_004129), *P. putida* GB-1 (NC_010322), *P. putida* W619 (NC_010501), *P. putida* KT2440 (NC_002947), *P. entomophila* L48 (NC_008027) , *P. putida* F1 (NC_009512), *P. fluorescens* SBW25 (NC_012660), *P. syringae pv. syringae* B728a (NC_007005) and *P .syringae pv. phaseolicola* 1448A (NC_005773). Analysis was performed using RAST.
(TIF)

Figure S11 Organisation and comparison of catabolic genes involved in phenylpropanoid pathway in *P. putida* CSV86 against *P. putida* F1 (NC_009512), *P. putida* W619 (NC_010501), *P. putida* KT2440 (NC_002947), *P.* syringae *pv. tomato str*. DC3000 (NC_004578) and *P. putida* GB-1 (NC_010322). Analysis was performed using RAST.
(TIF)

Figure S12 Organization and comparison of catabolic genes involved in homogentisate pathway in CSV86 against *P. putida* KT2440 (NC_002947), *P. putida* F1 (NC_009512), *P. fluorescens* Pf-5 (NC_004129), *P. putida* W619 (NC_010501), *P. fluorescens* SBW25 (NC_012660), *P. fluorescens* PfO-1 (NC_007492), *P. putida* GB-1 (NC_010322) and *P. entomophila* L48 (NC_008027). Analysis was performed using RAST.
(TIF)

Figure S13 Organisation and comparison of catabolic genes involved in copper resistance in *P. putida* CSV86 against *P. putida GB-1* (NC_010322), *P. putida* W619 (NC_010501), *P. entomophila* L48 (NC_008027), *P. putida* KT2440 (NC_002947), *P. putida* F1 (NC_009512), *P. mendocina* ymp (NC_009439) and *P. stutzeri* A1501 (NC_009434). Analysis was performed using RAST.
(TIF)

Figure S14 Progressive alignment between the draft genomes of *P. putida* CSV86 and the complete genome of *P. stutzeri* CCUG 29243 (NC_018028), *Pseudomonas* sp. ND6 plasmid pND6-1, complete sequence (NC_005244) and *P. putida* plasmid NAH7(NC_007926) and *P. fluorescens* strain PC20 plasmid pNAH20 (AY887963). Naphthalene degrading upper operon has been aligned with all the 4 genomes. Integrase coding genes has been highlighted by the black vertical bar. Colored blocks outline genome sequence that align to part of another genome, and is presumably homologous and internally free from genomic rearrangement (Locally Colinear Blocks or LCBs). The white colored area indicates regions with no alignment as they may probably contain sequences specific to that genome. The inverted blocks below the centre line indicate regions that align in the reverse complement (inverse) orientation and the height of the colored bars signify the nucleotide sequence similarity.
(TIF)

Figure S15 Progressive alignment between the draft genomes of *P. putida* CSV86 and the complete genome of *P. stutzeri* CCUG 29243 (NC_018028), *Pseudomonas* sp. ND6 plasmid pND6-1 (NC_005244) and *Pseudomonas putida* plasmid NAH7(NC_007926) and *P. fluorescens* strain PC20 plasmid pNAH20 (AY887963) using MAUVE software. Naphthalene degrading lower operon has been aligned with all the 4 genomes. Transposase coding genes has been highlighted by the black vertical bar.
(TIF)

Figure S16 Output image of GC Profile software using nucleotide sequence of contig 105 having naphthalene degradation upper pathway gene. The arrow indicates the segmentation point 1 from where the GC content varies as compared to rest of the contig sequence. The GC content of 1–90554 bp region is 63.8, whereas the region after the segmentation point i.e. 90555–100230 bp has GC content of 51.64 (segmentation strength-258.32).
(TIF)

Table S1 Summary of *P. putida* CSV86 draft genome compared with other complete genome of *Pseudomonas* spp. available in KEGG database.
(DOC)

Table S2 Pathways present in *P. putida* CSV86 genome based on NCBI PGAAP annotation.
(DOCX)

Table S3 Percentage homology of Naphthalene upper and lower operon pathway proteins in *P. putida* CSV86 with homologous proteins of 4 *Pseudomonas* species reported for naphthalene degradation capability.
(DOCX)

Table S4 Heavy metal resistance genes identified in *P. putida* CSV86 genome annotation in RAST and their percentage homology with the closest respective gene.
(DOCX)

Table S5 Mobile genetic elements present in *P. putida* CSV86 genome located using IS finder.
(DOCX)
