# Supplementary figures and images for: Pseudomonas putida CSV86: A Candidate Genome for Genetic Bioaugmentation
Source: PLoS One. 2014 Jan 24;9(1):e84000. doi: 10.1371/journal.pone.0084000 (PMC3901652; doi:10.1371/journal.pone.0084000)

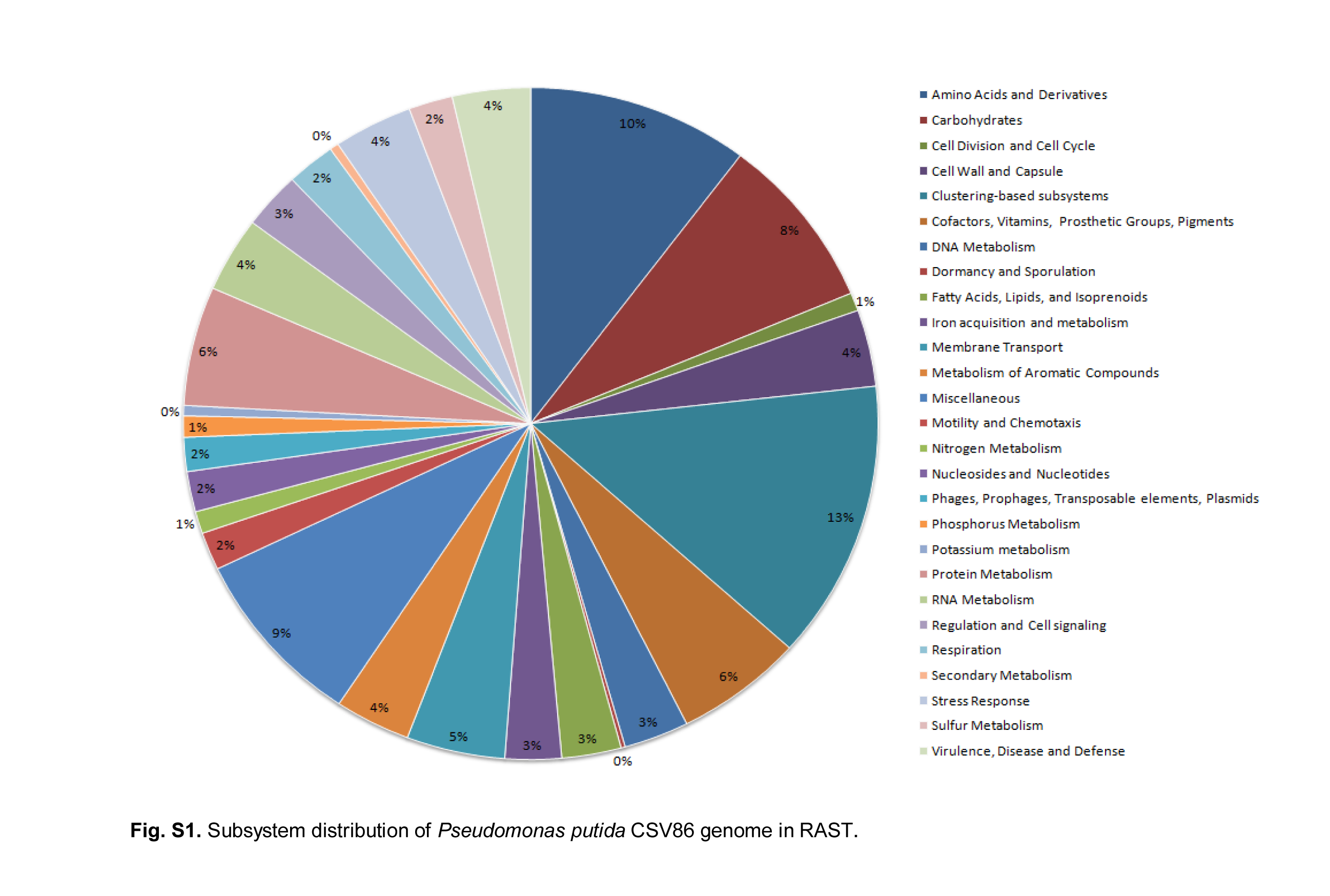

Supplement: Figure S1 — Subsystem distribution of Pseudomonas putida CSV86 genome in RAST. (TIF) [file pone.0084000.s001.tif]

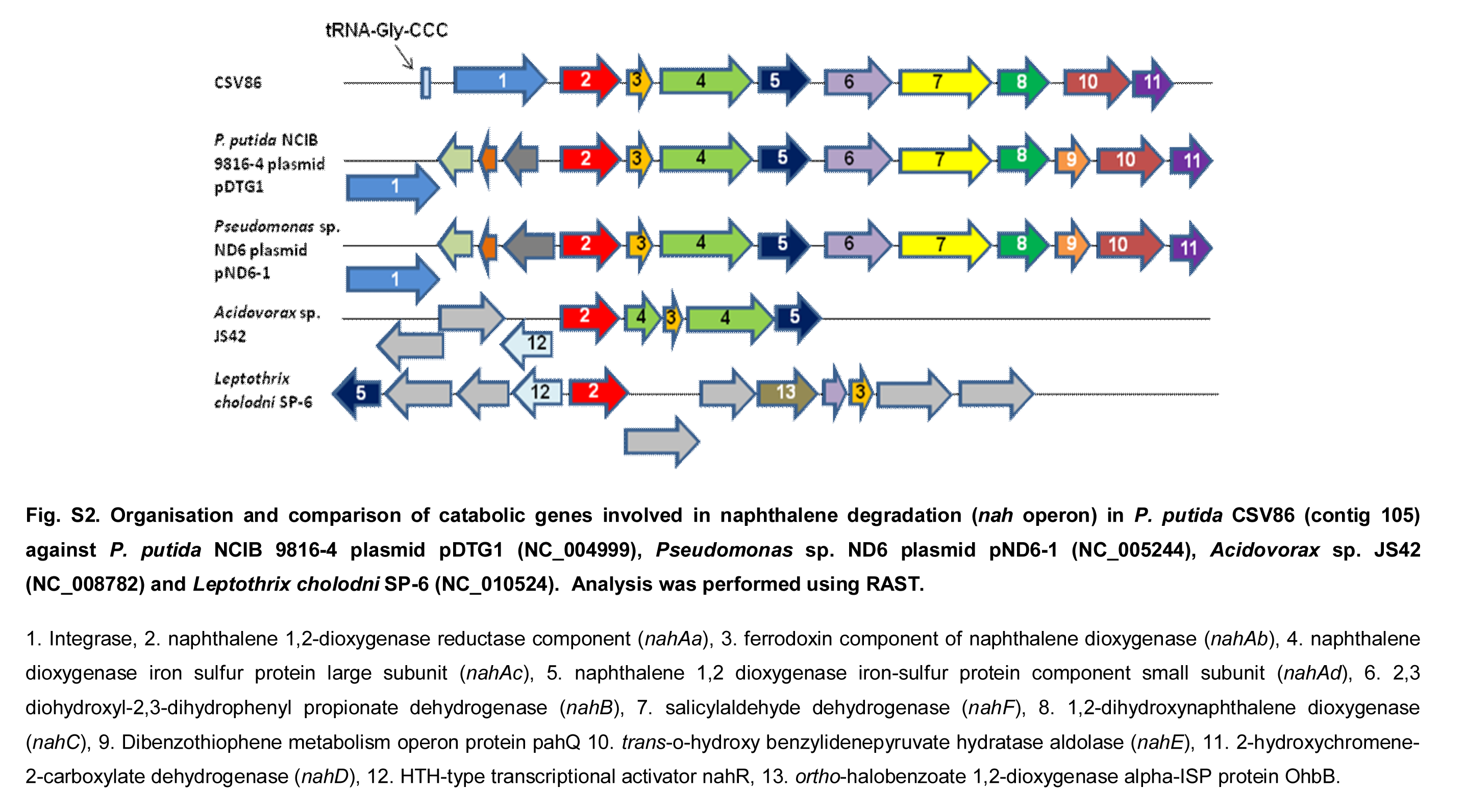

Supplement: Figure S2 — Organisation and comparison of catabolic genes involved in naphthalene degradation (nah operon) in P. putida CSV86 (contig 105) against P. putida NCIB 9816-4 plasmid pDTG1 (NC_004999), Pseudomonas sp. ND6 plasmid pND6-1 (NC_005244), Acidovorax sp. JS42 (NC_008782) and Leptothrix cholodni SP-6 (NC_010524). Analysis was performed using RAST. (TIF) [file pone.0084000.s002.tif]

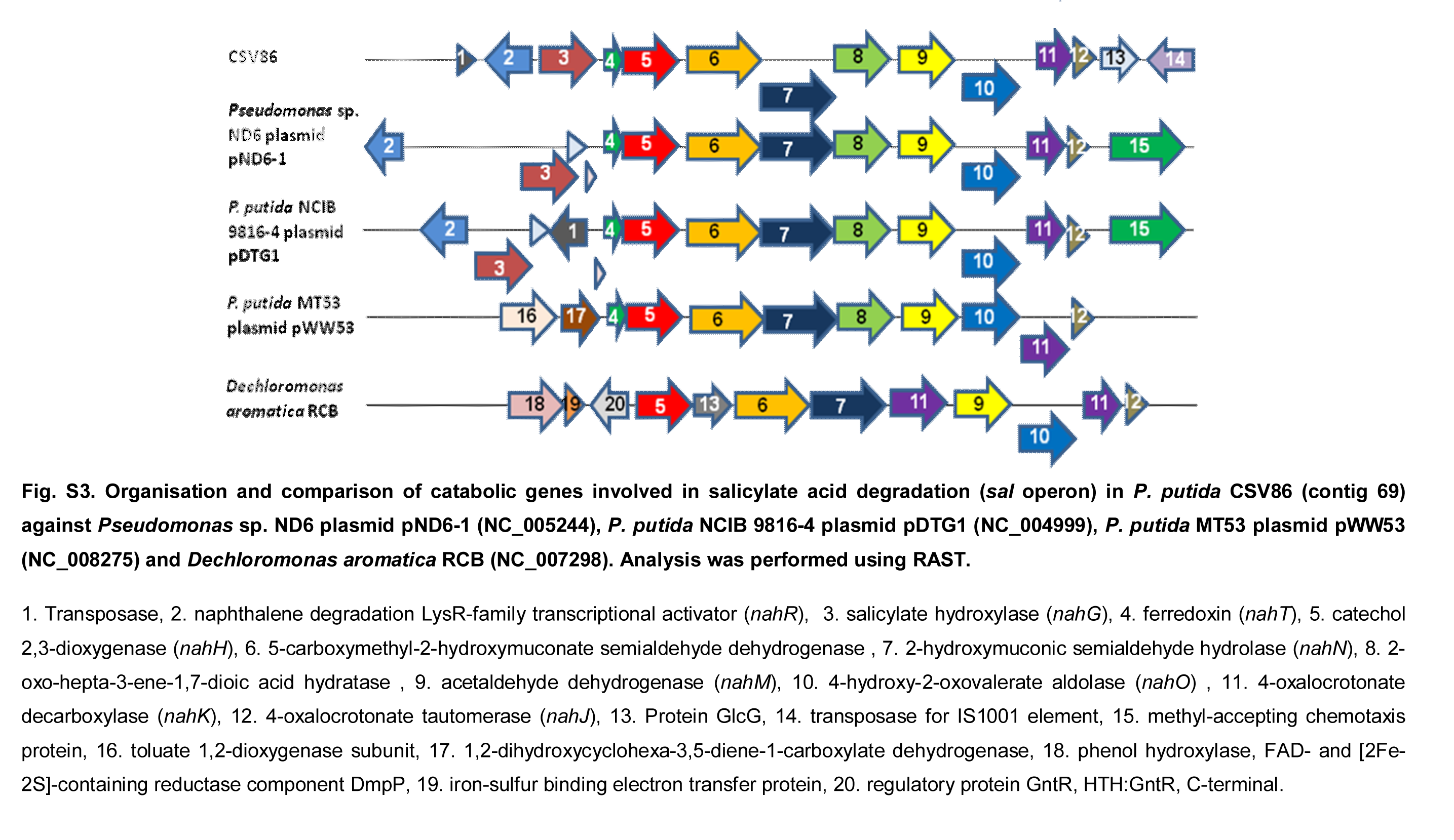

Supplement: Figure S3 — Organisation and comparison of catabolic genes involved in salicylate acid degradation (sal operon) in P. putida CSV86 (contig 69) against Pseudomonas sp. ND6 plasmid pND6-1 (NC_005244), P. putida NCIB 9816-4 plasmid pDTG1 (NC_004999), P. putida MT53 plasmid pWW53 (NC_008275) and Dechloromonas aromatica RCB (NC_007298). Analysis was performed using RAST. (TIF) [file pone.0084000.s003.tif]

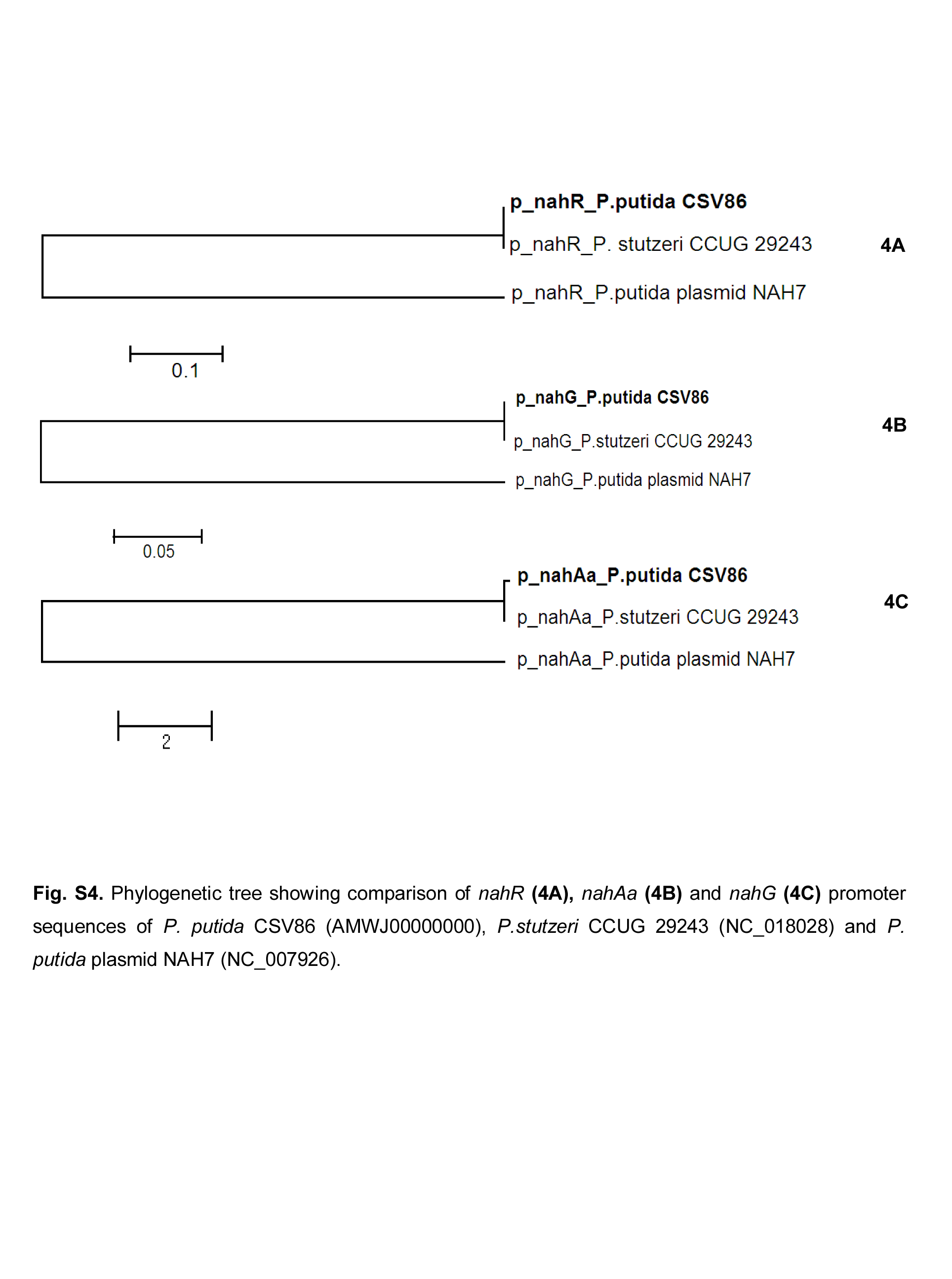

Supplement: Figure S4 — Phylogenetic tree showing comparison of nahR (4A), nahAa (4B) and nahG (4C) promoter sequences of P. putida CSV86 (AMWJ00000000), P.stutzeri CCUG 29243 (NC_018028) and P. putida plasmid NAH7 (NC_007926). (TIF) [file pone.0084000.s004.tif]

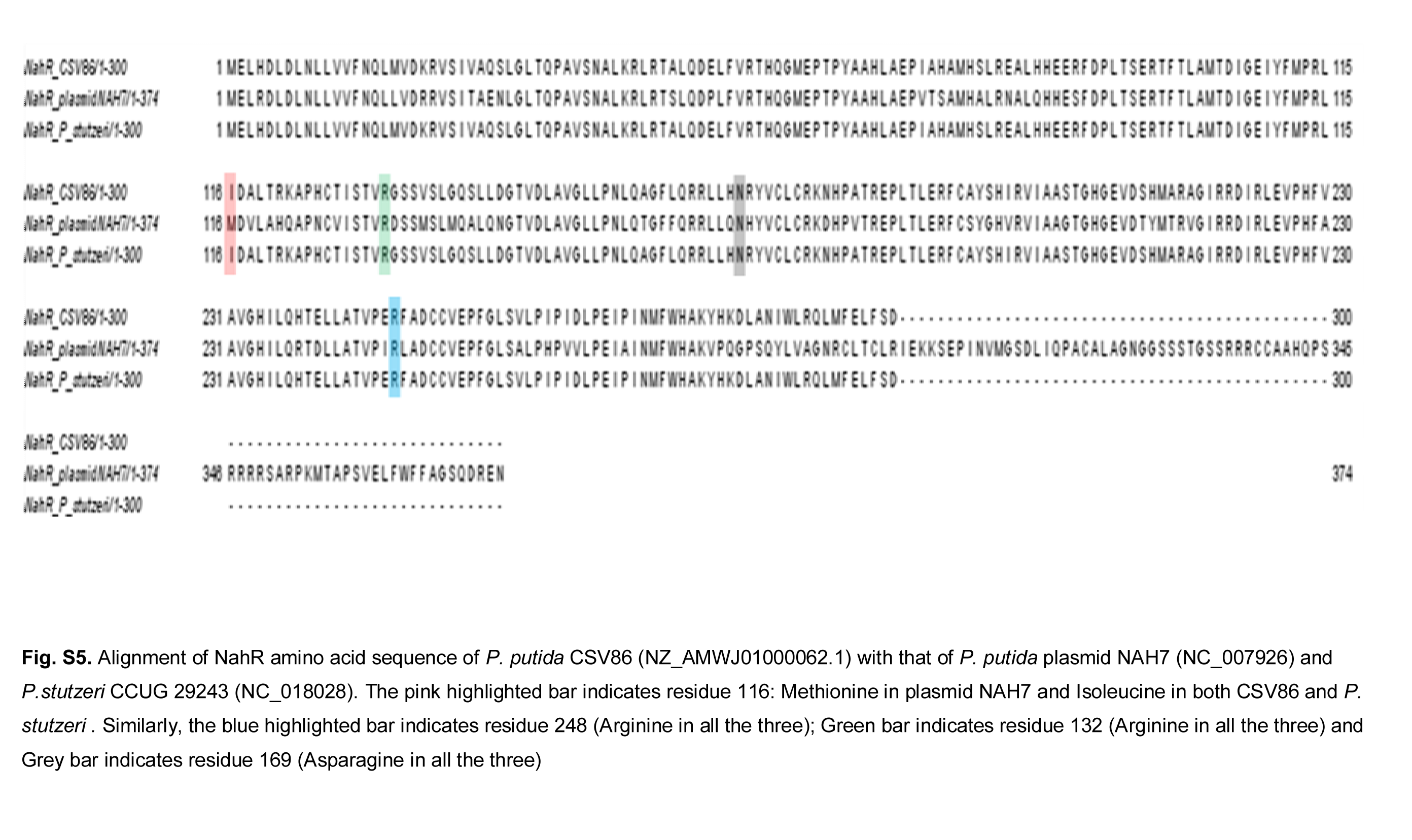

Supplement: Figure S5 — Alignment of NahR amino acid sequence of P. putida CSV86 (NZ_AMWJ01000062.1) with that of P. putida plasmid NAH7 (NC_007926) and P.stutzeri CCUG 29243 (NC_018028). The pink highlighted bar indicates residue 116: Methionine in plasmid NAH7 and Isoleucine in both CSV86 and P. stutzeri. Similarly, the blue highlighted bar indicates residue 248 (Arginine in all the three); Green bar indicates residue 132 (Arginine in all the three) and Grey bar indicates residue 169 (Asparagine in all the three). (TIF) [file pone.0084000.s005.tif]

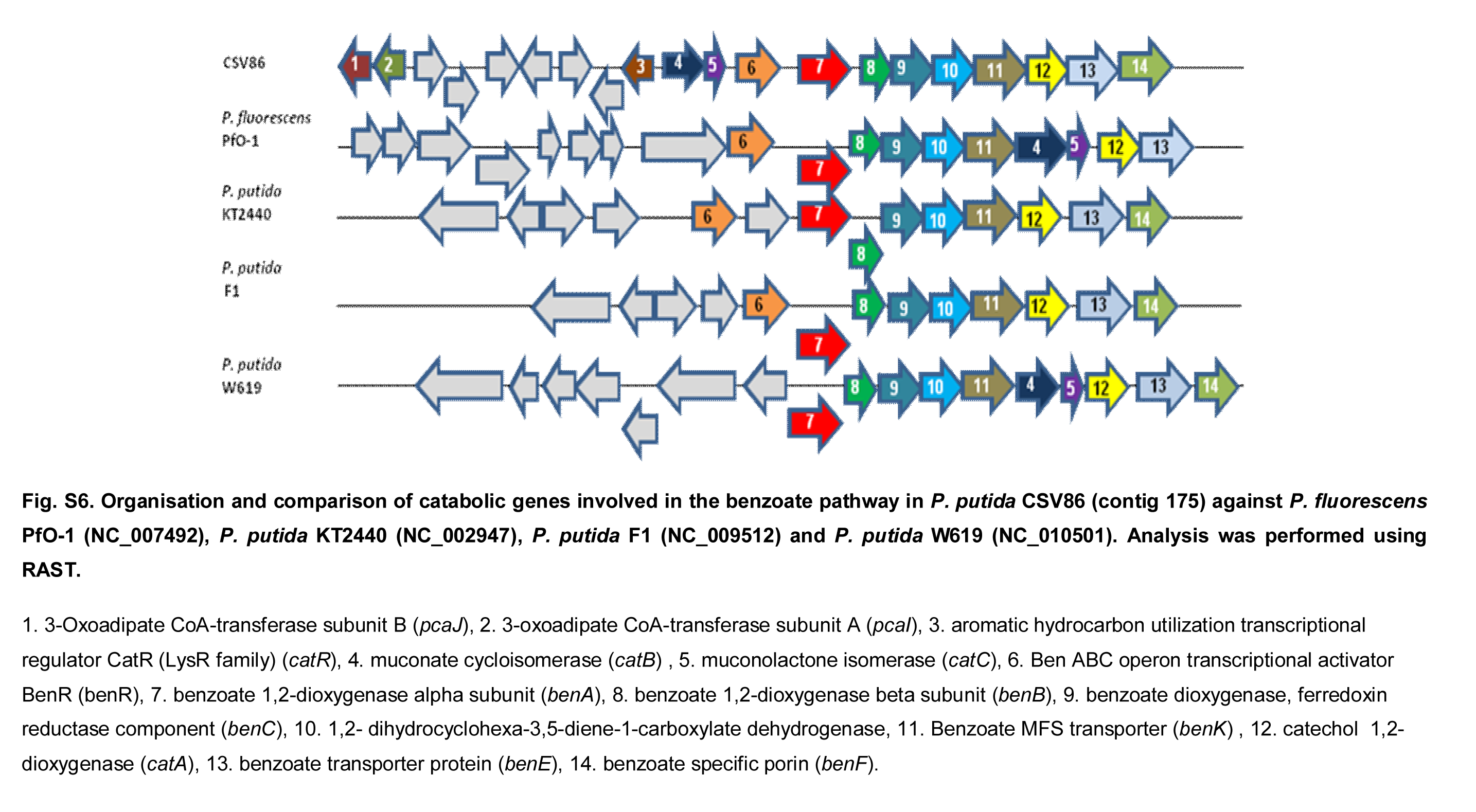

Supplement: Figure S6 — Organisation and comparison of catabolic genes involved in the benzoate pathway in P. putida CSV86 (contig 175) against P. fluorescens PfO-1 (NC_007492), P. putida KT2440 (NC_002947), P. putida F1 (NC_009512) and P. putida W619 (NC_010501). Analysis was performed using RAST. (TIF) [file pone.0084000.s006.tif]

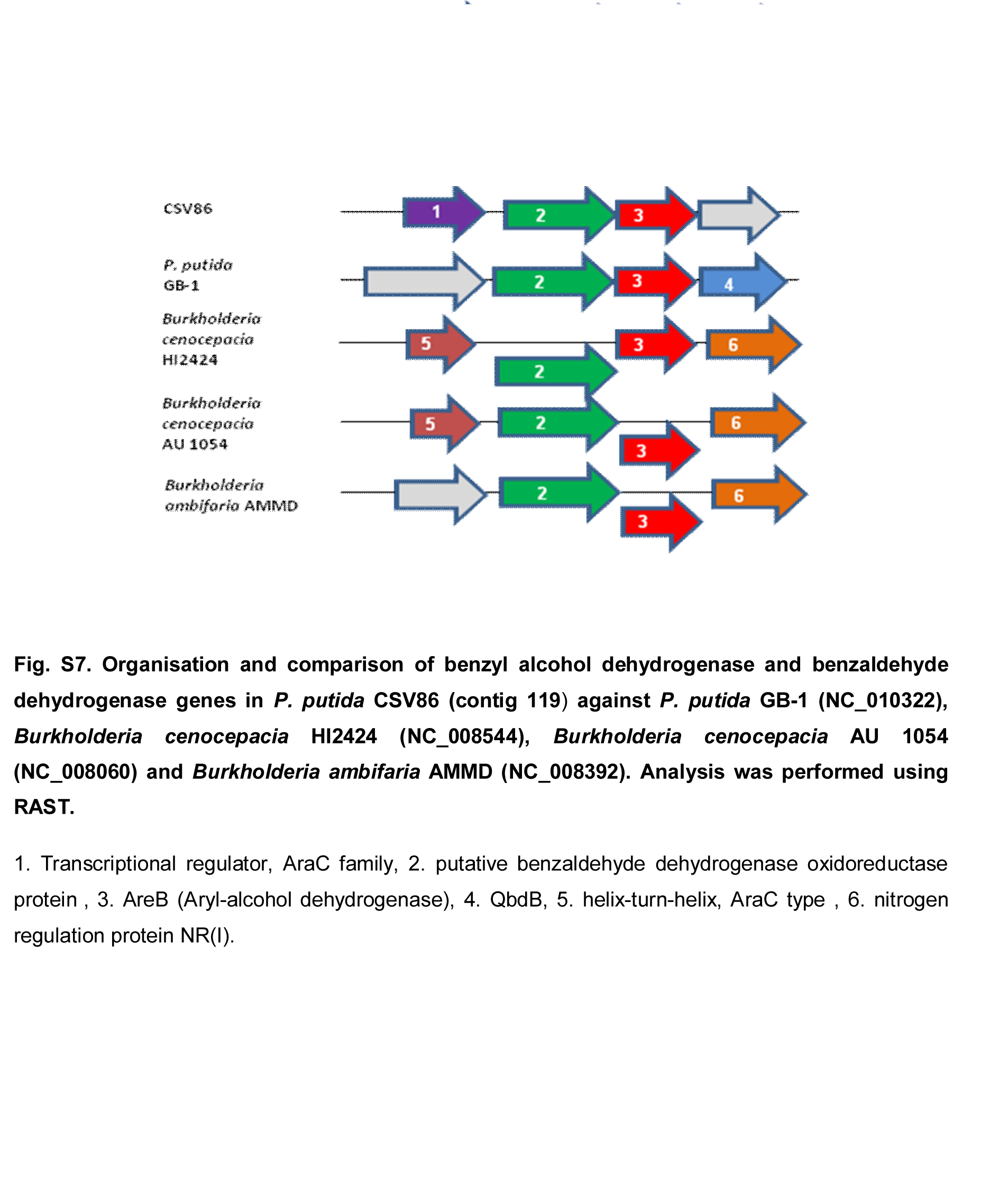

Supplement: Figure S7 — Organisation and comparison of benzyl alcohol dehydrogenase and benzaldehyde dehydrogenase genes in P. putida CSV86 (contig 119) against P. putida GB-1 (NC_010322), Burkholderia cenocepacia HI2424 (NC_008544), Burkholderia cenocepacia AU 1054 (NC_008060) and Burkholderia ambifaria AMMD (NC_008392). Analysis was performed using RAST. (TIF) [file pone.0084000.s007.tif]

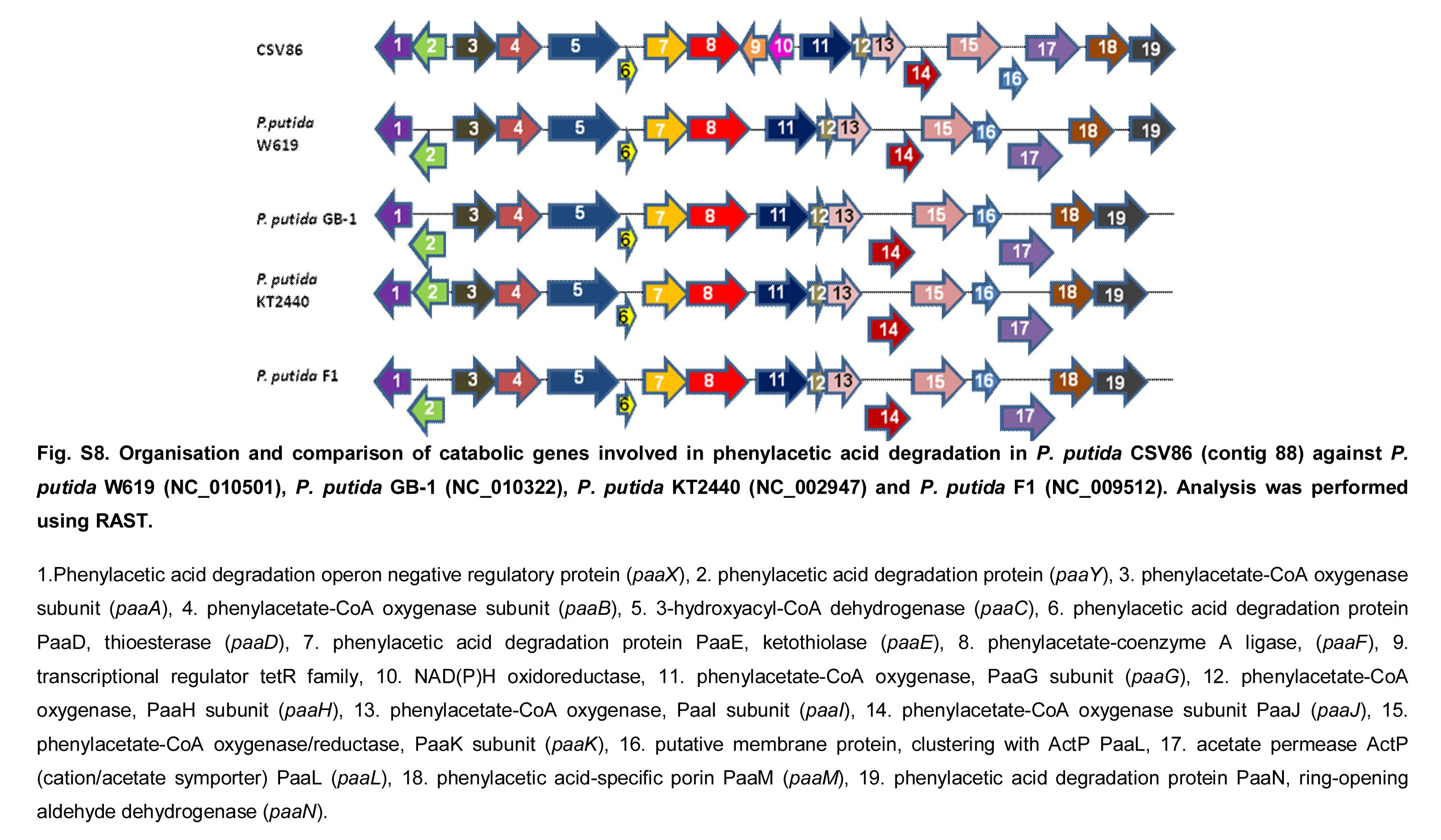

Supplement: Figure S8 — Organisation and comparison of catabolic genes involved in phenylacetic acid degradation in P. putida CSV86 (contig 88) against P. putida W619 (NC_010501), P. putida GB-1 (NC_010322), P. putida KT2440 (NC_002947) and P. putida F1 (NC_009512). Analysis was performed using RAST. (TIF) [file pone.0084000.s008.tif]

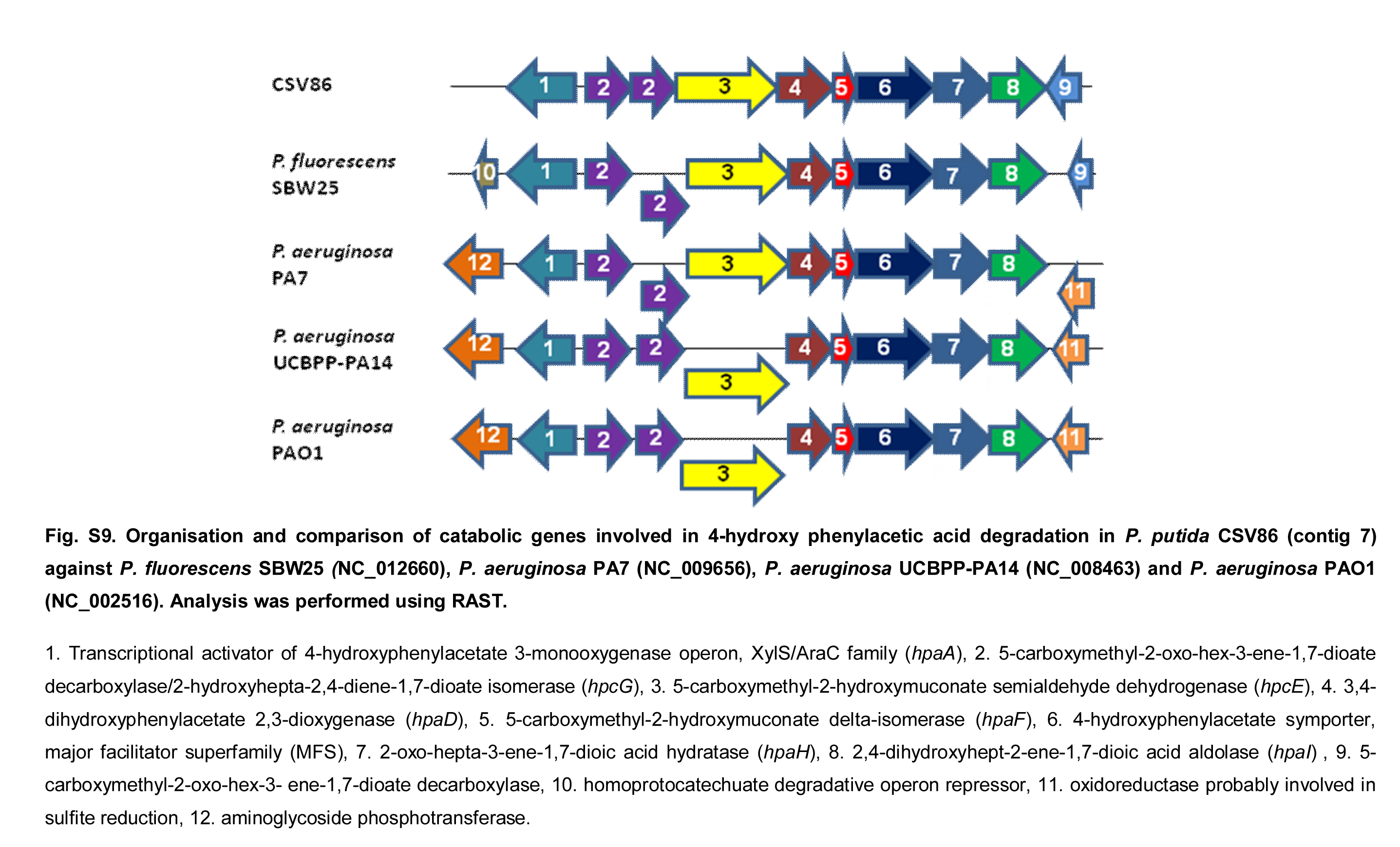

Supplement: Figure S9 — Organisation and comparison of catabolic genes involved in 4-hydroxy phenylacetic acid degradation in P. putida CSV86 (contig 7) against P. fluorescens SBW25 (NC_012660), P. aeruginosa PA7 (NC_009656), P. aeruginosa UCBPP-PA14 (NC_008463) and P. aeruginosa PAO1 (NC_002516). Analysis was performed using RAST. (TIF) [file pone.0084000.s009.tif]

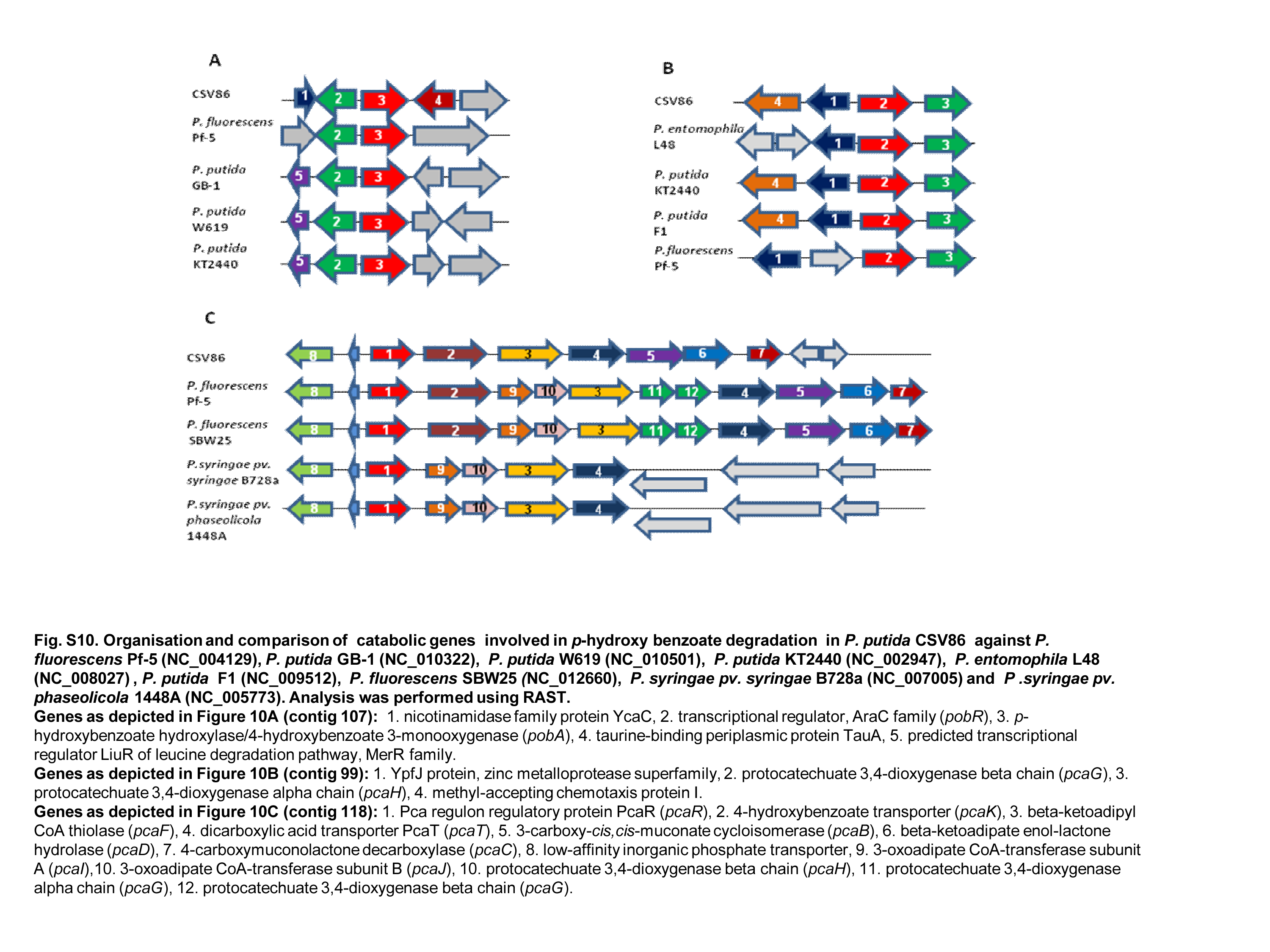

Supplement: Figure S10 — Organisation and comparison of catabolic genes involved in phydroxy benzoate degradation in P. putida CSV86 against P. fluorescens Pf-5 (NC_004129), P. putida GB-1 (NC_010322), P. putida W619 (NC_010501), P. putida KT2440 (NC_002947), P. entomophila L48 (NC_008027) , P. putida F1 (NC_009512), P. fluorescens SBW25 (NC_012660), P. syringae pv. syringae B728a (NC_007005) and P .syringae pv. phaseolicola 1448A (NC_005773). Analysis was performed using RAST. (TIF) [file pone.0084000.s010.tif]

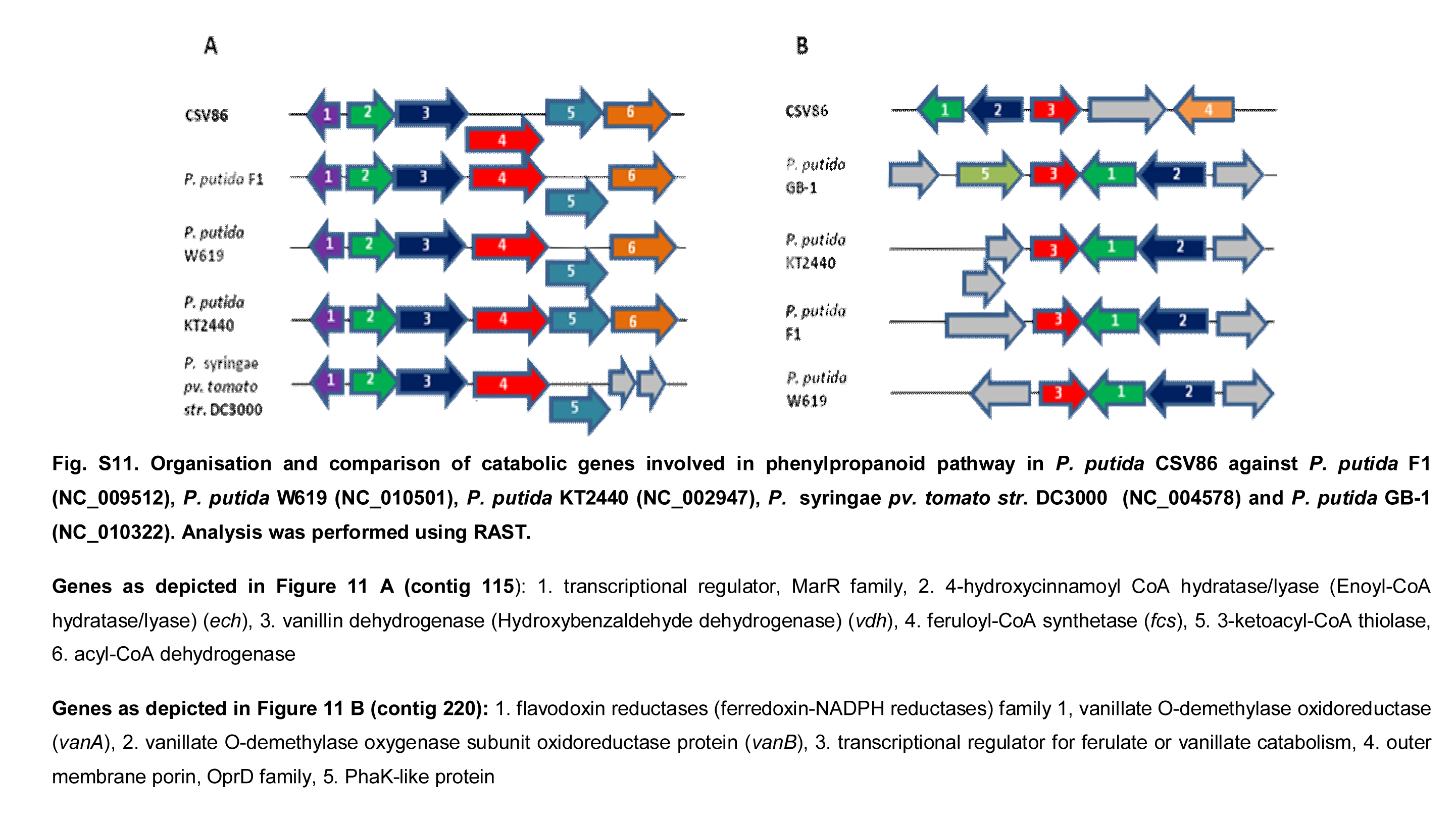

Supplement: Figure S11 — Organisation and comparison of catabolic genes involved in phenylpropanoid pathway in P. putida CSV86 against P. putida F1 (NC_009512), P. putida W619 (NC_010501), P. putida KT2440 (NC_002947), P. syringae pv. tomato str. DC3000 (NC_004578) and P. putida GB-1 (NC_010322). Analysis was performed using RAST. (TIF) [file pone.0084000.s011.tif]

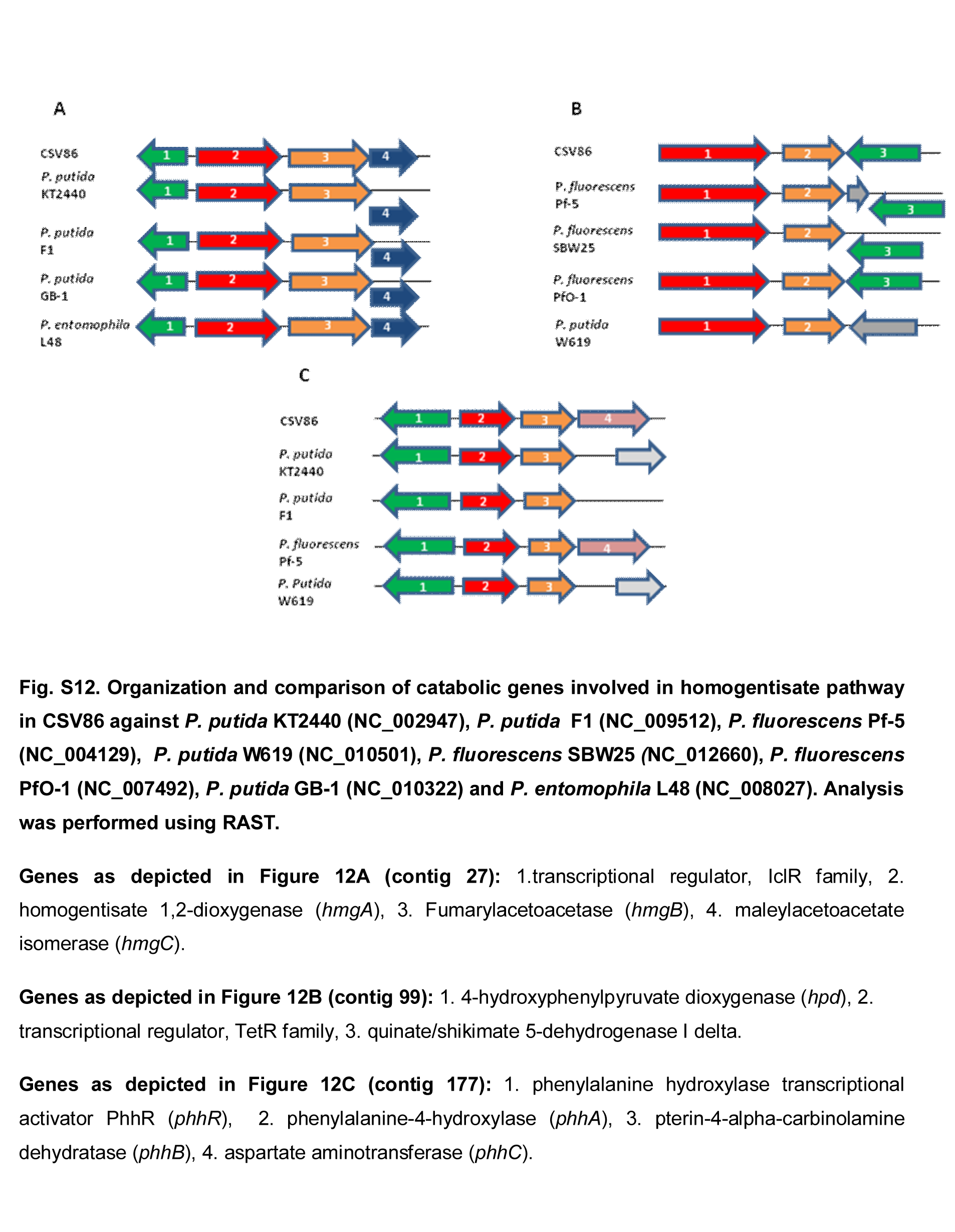

Supplement: Figure S12 — Organization and comparison of catabolic genes involved in homogentisate pathway in CSV86 against P. putida KT2440 (NC_002947), P. putida F1 (NC_009512), P. fluorescens Pf-5 (NC_004129), P. putida W619 (NC_010501), P. fluorescens SBW25 (NC_012660), P. fluorescens PfO-1 (NC_007492), P. putida GB-1 (NC_010322) and P. entomophila L48 (NC_008027). Analysis was performed using RAST. (TIF) [file pone.0084000.s012.tif]

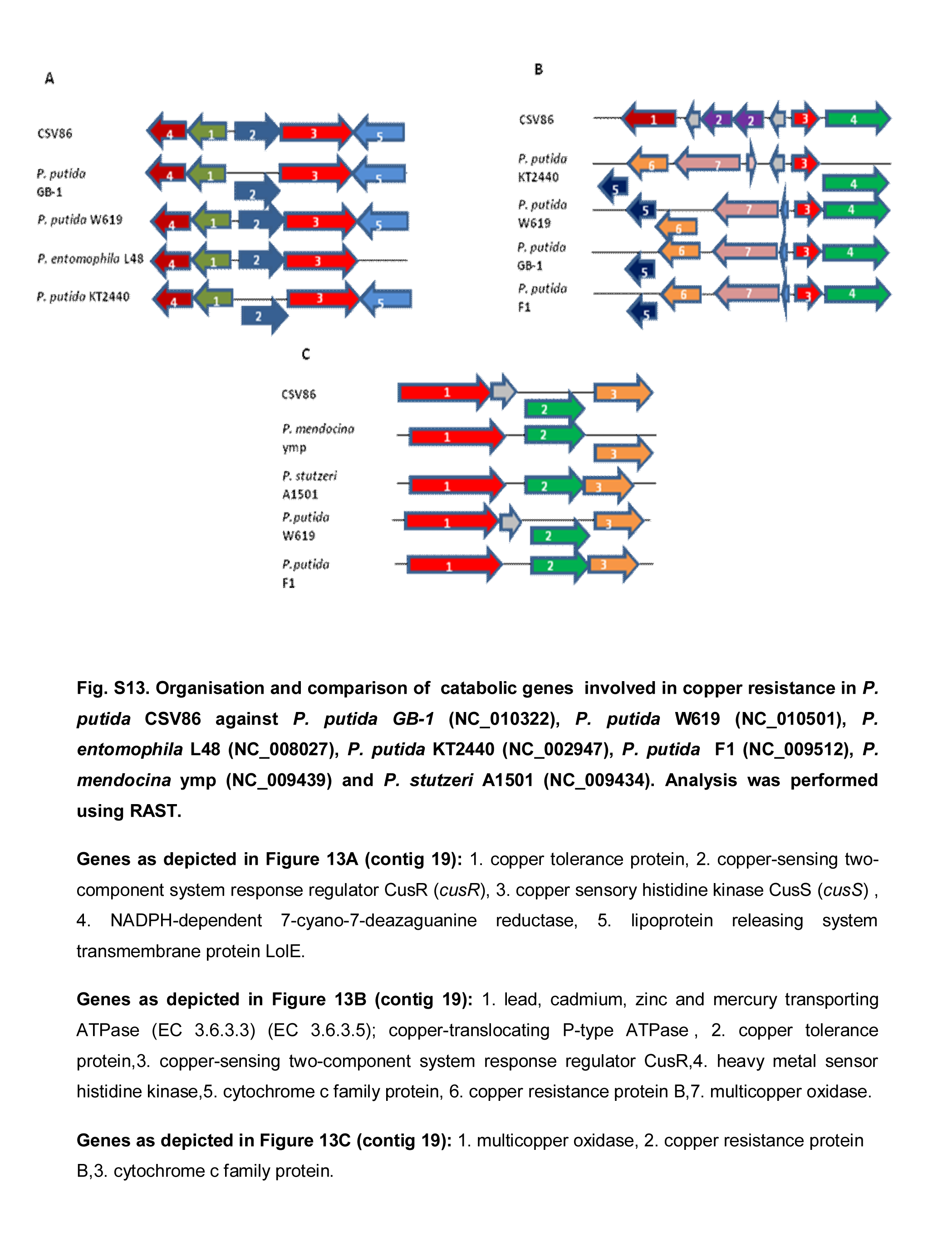

Supplement: Figure S13 — Organisation and comparison of catabolic genes involved in copper resistance in P. putida CSV86 against P. putida GB-1 (NC_010322), P. putida W619 (NC_010501), P. entomophila L48 (NC_008027), P. putida KT2440 (NC_002947), P. putida F1 (NC_009512), P. mendocina ymp (NC_009439) and P. stutzeri A1501 (NC_009434). Analysis was performed using RAST. (TIF) [file pone.0084000.s013.tif]

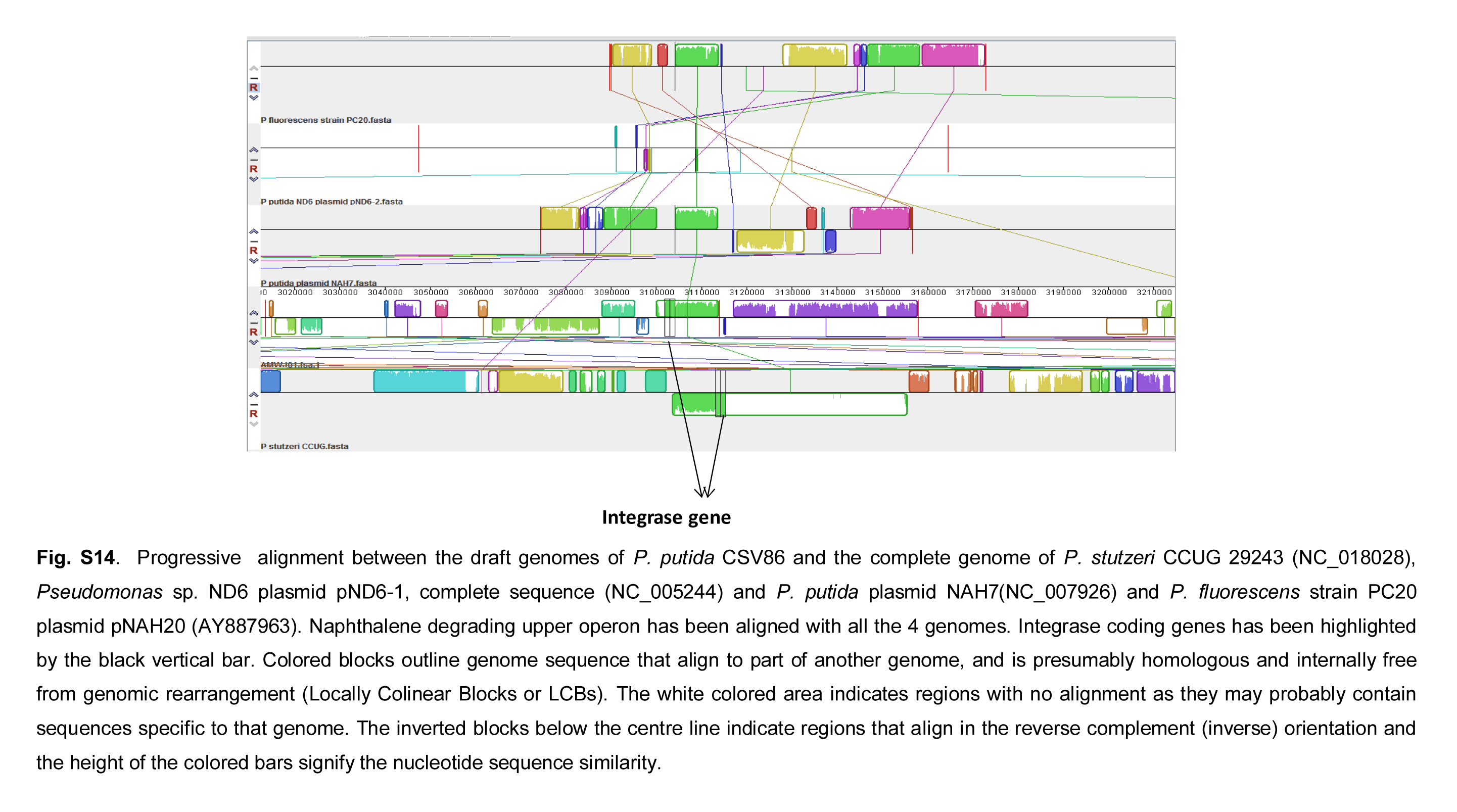

Supplement: Figure S14 — Progressive alignment between the draft genomes of P. putida CSV86 and the complete genome of P. stutzeri CCUG 29243 (NC_018028), Pseudomonas sp. ND6 plasmid pND6-1, complete sequence (NC_005244) and P. putida plasmid NAH7(NC_007926) and P. fluorescens strain PC20 plasmid pNAH20 (AY887963). Naphthalene degrading upper operon has been aligned with all the 4 genomes. Integrase coding genes has been highlighted by the black vertical bar. Colored blocks outline genome sequence that align to part of another genome, and is presumably homologous and internally free from genomic rearrangement (Locally Colinear Blocks or LCBs). The white colored area indicates regions with no alignment as they may probably contain sequences specific to that genome. The inverted blocks below the centre line indicate regions that align in the reverse complement (inverse) orientation and the height of the colored bars signify the nucleotide sequence similarity. (TIF) [file pone.0084000.s014.tif]

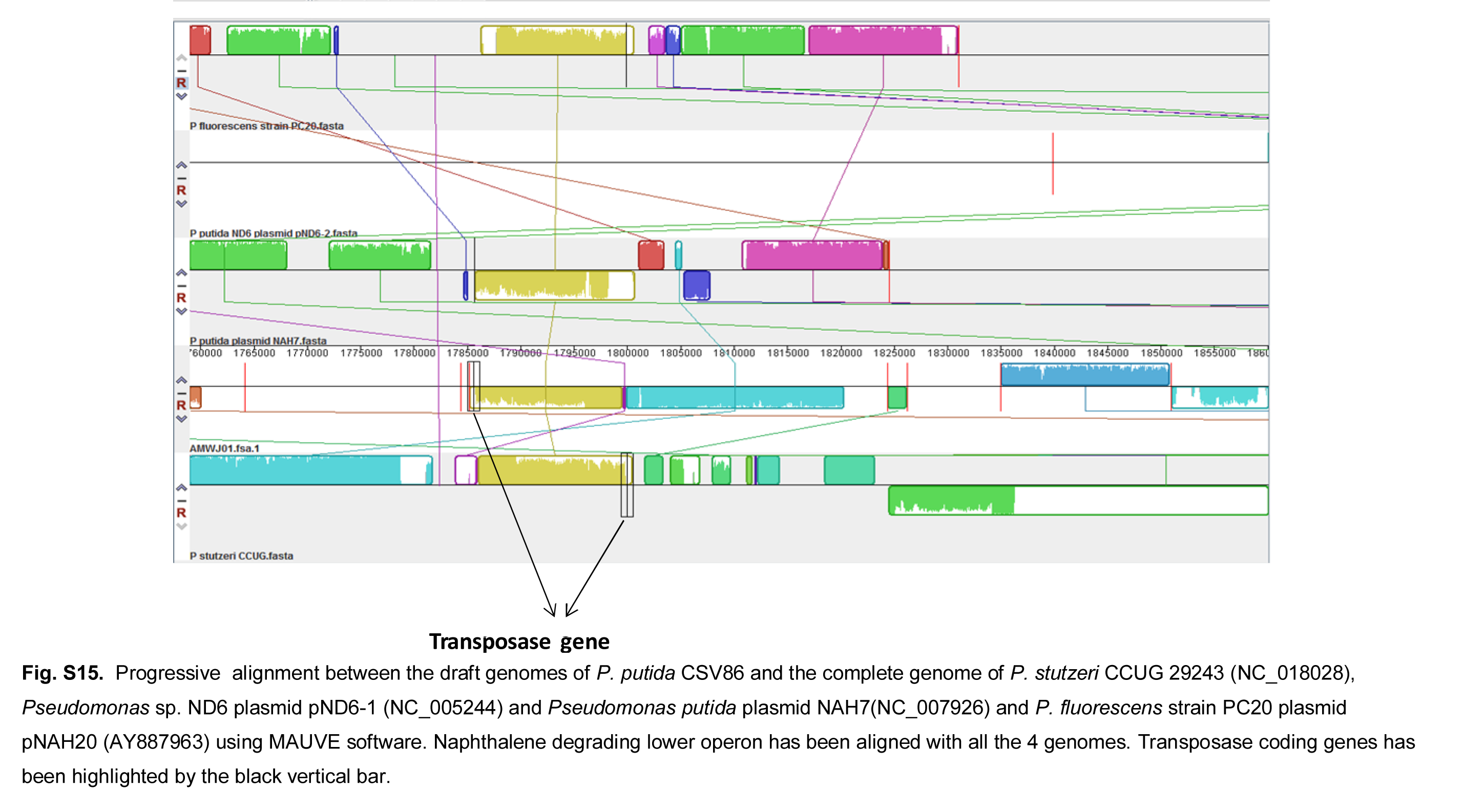

Supplement: Figure S15 — Progressive alignment between the draft genomes of P. putida CSV86 and the complete genome of P. stutzeri CCUG 29243 (NC_018028), Pseudomonas sp. ND6 plasmid pND6-1 (NC_005244) and Pseudomonas putida plasmid NAH7(NC_007926) and P. fluorescens strain PC20 plasmid pNAH20 (AY887963) using MAUVE software. Naphthalene degrading lower operon has been aligned with all the 4 genomes. Transposase coding genes has been highlighted by the black vertical bar. (TIF) [file pone.0084000.s015.tif]

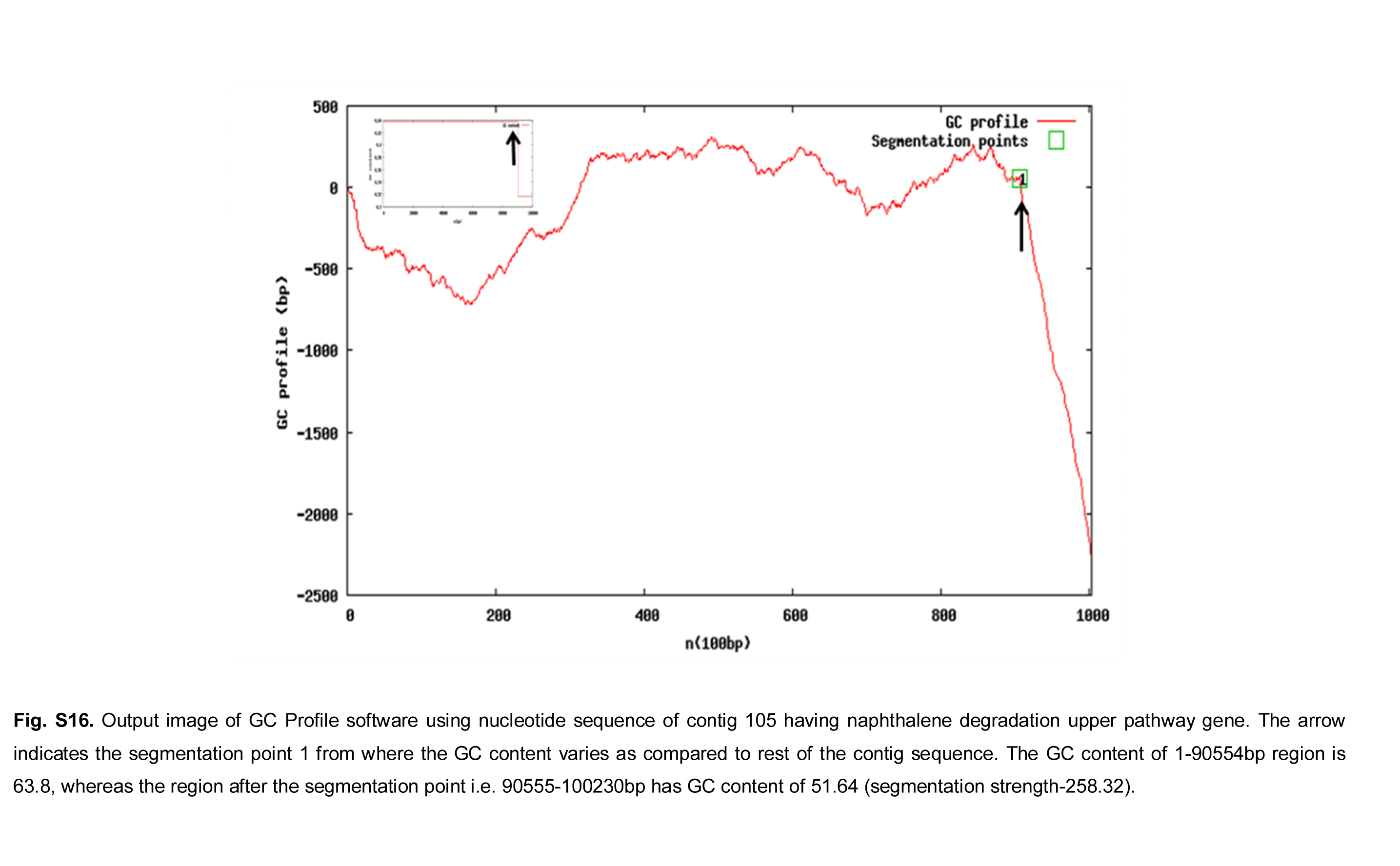

Supplement: Figure S16 — Output image of GC Profile software using nucleotide sequence of contig 105 having naphthalene degradation upper pathway gene. The arrow indicates the segmentation point 1 from where the GC content varies as compared to rest of the contig sequence. The GC content of 1–90554 bp region is 63.8, whereas the region after the segmentation point i.e. 90555–100230 bp has GC content of 51.64 (segmentation strength-258.32). (TIF) [file pone.0084000.s016.tif]
