# Supplementary material for: Pseudomonas putida CSV86: A Candidate Genome for Genetic Bioaugmentation
Source: PLoS One. 2014 Jan 24;9(1):e84000. doi: 10.1371/journal.pone.0084000 (PMC3901652; doi:10.1371/journal.pone.0084000)
Supplement: Table S1 — Summary of P. putida CSV86 draft genome compared with other complete genome of Pseudomonas spp. available in KEGG database. (DOC) [file pone.0084000.s017.doc]

**Table S1.** Summary of *P. putida* CSV86 draft genome compared with other complete genome of *Pseudomonas* spp. available in KEGG database

| **S. No.** | **Sample** | **Accession no.** | **Length (bp)** | **Total genes** | **Proteins coding** | **COG assigned genes** |
| --- | --- | --- | --- | --- | --- | --- |
|  | ***P.* *putida* CSV86** | **AMWJ00000000** | **6,469,780** | **5900** | **5836** | **4642** |
|  | *P.* *putida* S16 | NC_015733 | 5984790 | 5369 | 5218 | 4820 |
|  | *P. putida* F1 | NC_009512 | 5959964 | 5403 | 5250 | 4338 |
|  | *P. putida* GB-1 | NC_010322 | 6078430 | 5529 | 5408 | 4435 |
|  | *P. putida* KT2440 | NC_002947 | 6181863 | 5516 | 5350 | 4407 |
|  | *P.* *putida* W619 | NC_010501 | 5774330 | 5309 | 5182 | 4262 |
|  | *P. putida* BIRD-1 | NC_017530 | 5731541 | 5046 | 4960 | - |
|  | *P. putida* HB3267 | NC_019905 | 5956110 | 5478 | 5386 | 4262 |
|  | *P. putida* ND6 | NC_017986 | 6304310 | 6484 | 6391 | - |
|  | *P. putida* UW4 | NC_019670 | 6183388 | 5537 | 5423 | 2969 |
|  | *P.* *entomophila* L48 | NC_008027 | 5888780 | 5275 | 5134 | 4159 |
|  | *P.* *aeruginosa* LESB58 | NC_011770 | 6601757 | 6061 | 5925 | 4820 |
|  | *P.* *aeruginosa* PA7 | NC_009656 | 6588339 | 6369 | 6286 | 4860 |
|  | *P.* *aeruginosa* PAO1 | NC_002516 | 6264404 | 5682 | 5571 | 4725 |
|  | *P.* *aeruginosa* UCBPP-PA14 | NC_008463 | 6537648 | 5977 | 5892 | 4843 |
|  | *P. aeruginosa* B136*-33* | NC_020912 | 6421010 | 5904 | 5828 | 4803 |
|  | *P. aeruginosa* DK2 | NC_018080 | 6402658 | 5960 | 5884 | 4776 |
|  | *P. aeruginosa* M18 | NC_017548 | 6327754 | 5770 | 5684 | - |
|  | *P. aeruginosa* NCGM2.S1 | NC_017549 | 6764661 | 6,357 | 6268 | - |
|  | *P.* *stutzeri* A1501 | NC_009434 | 4567418 | 4209 | 4127 | 3474 |
|  | *P. stutzeri* RCH2 | NC_019936 | 4600489 | 4405 | 4265 | - |
|  | *P. stutzeri* DSM4166 | NC_017532 | 4689946 | 4373 | 4301 | - |
|  | *P. stutzeri* CCUG29243 | NC_018028 | 4709064 | 4374 | 4300 | 3558 |
|  | *P. stutzeri* DSM10701 | NC_018177 | 4174118 | 3888 | 3815 | 3228 |
|  | *P. stutzeri* ATCC17588 | NC_015740 | 4547930 | 4314 | 4217 | - |
|  | *P.* *fluorescens* Pf0-1 | NC_007492 | 6438405 | 5829 | 5722 | 4733 |
|  | *P.* *fluorescens* SBW25 | NC_012660 | 6722539 | 6106 | 5921 | - |
|  | *P. fluorescens* A506 | NC_017911 | 6019547 | 5428 | 5334 | - |
|  | *P. fluorescens* F113 | NC_016830 | 6845832 | 5962 | 5862 | - |
|  | *P.* *protegens* Pf-5 | NC_004129 | 7074893 | 6273 | 6108 | 5061 |
|  | *P.* *syringae* pv. phaseolicola 1448A | NC_005773 | 6112448 | 5437 | 5172 | 4156 |
|  | *P.* *syringae* B728a | NC_007005 | 6093698 | 5220 | 5089 | 4178 |
|  | *P.* *syringae* pv. tomato str. DC3000 | NC_004578 | 6538260 | 5842 | 5619 | 4366 |
|  | *P. mendocina* NK-01 | NC_015410 | 5434353 | 5035 | 4958 | - |
|  | *P.* *mendocina* ymp | NC_009439 | 5072807 | 4704 | 4594 | 3841 |
|  | *P. denitrificans* ATCC13867 | NC_020829 | 5696307 | 5135 | 5056 | 4285 |
|  | *P. poae RE**1-1-14 | NC_020209 | 5512241 | 4877 | 4796 | 3959 |
|  | *P. brassicacearum* NFM421 | NC_015379 | 6843248 | 6176 | 6095 | - |
|  | *P. fulva* 12*-*X | NC_015556 | 4920769 | 4575 | 4461 | - |
