# Supplementary material for: Pseudomonas putida CSV86: A Candidate Genome for Genetic Bioaugmentation
Source: PLoS One. 2014 Jan 24;9(1):e84000. doi: 10.1371/journal.pone.0084000 (PMC3901652; doi:10.1371/journal.pone.0084000)
Supplement: Table S2 — Pathways present in P. putida CSV86 genome based on NCBI PGAAP annotation. (DOCX) [file pone.0084000.s018.docx]

**Table S2.** Pathways present in *P. putida* CSV86 genome based on NCBI PGAAP annotation

| **Pathway**  **(Contig No.)** | **Gene** | **Locus** |
| --- | --- | --- |
| **Naphthalene**  **upper**  **pathway**  **(105)** | naphthalene 1,2-dioxygenase reductase component, nahAa | CSV86_14319 |
|  | ferredoxin component naphthalene dioxygenase, nahAb | CSV86_14324 |
|  | napthalene dioxygenase iron sulfur protein large subunit, nahAc | CSV86_14334 |
|  | naphthalene 1,2-dioxygenase iron sulfur protein component small subunit, nahAd | CSV86_14339 |
|  | 2,3-dihydroxy-2,3-dihydrophenylpropionate dehydrogenase, nahB | CSV86_14344 |
|  | salicylaldehyde dehydrogenase NahF | CSV86_14349 |
|  | 1,2-dihydroxynaphthalene dioxygenase, nahC | CSV86_14354 |
|  | trans-o-hydroxybenzylidenepyruvate hydratase-aldolase NahE | CSV86_14359 |
|  | 2-hydroxychromene-2-carboxylate isomerase, nahD | CSV86_14364 |
| **Salicylate**  **lower**  **pathway**  **(69)** | naphthalene degradation LysR-family transcriptional activator | CSV86_08336 |
|  | salicylate hydroxylase, nahG | CSV86_08341 |
|  | chloroplast-type ferredoxin, nahT | CSV86_08346 |
|  | catechol 2,3-dioxygenase, nahH | CSV86_08351 |
|  | 2-hydroxymuconic semialdehyde dehydrogenase, nahI | CSV86_08356 |
|  | 2-hydroxymuconic semialdehyde hydrolase, nahN | CSV86_08361 |
|  | 2-oxopent-4-enoate hydratase, nahL | CSV86_08366 |
|  | acetaldehyde dehydrogenase, nahM | CSV86_08371 |
|  | 4-hyroxy-2-oxovalerate/4-hydroxy-2-oxopentanoic acid aldolase, nahO | CSV86_08376 |
|  | 4-oxalocrotonate decarboxylase, nahK | CSV86_08381 |
|  | 4-oxalocrotonate tautomerase, nahJ | CSV86_08386 |
| **Benzoate pathway**  **(103, 175, 116, 118)** | AraC family transcriptional regulator | CSV86_23504 |
|  | Benzoate 1,2-dioxygenase, alpha subunit, benA | CSV86_23499 |
|  | 2-chlorobenzoate 1,2-dioxygenase | CSV86_23494 |
|  | benzoate 1,2-dioxygenase electron transfer protein, benC | CSV86_23489 |
|  | 1,6-dihydroxycyclohexa-2,4-diene-1-carboxylate dehydrogenase, benD | CSV86_23484 |
|  | major facilitator transporter | CSV86_23479 |
|  | benzoate transporter | CSV86_23469 |
|  | outer membrane porin | CSV86_23464 |
|  | putative transcriptional regulator | CSV86_13680 |
|  | intradiol ring-cleavage dioxygenase | CSV86_23474 |
|  | muconate and chloromuconate cycloisomerase, catB | CSV86_23514 |
|  | muconolactone delta-isomerase, catC | CSV86_23509 |
|  | 3-oxoadipate enol-lactonase/ 4-carboxymuconolactone decarboxylase, pcaD | CSV86_16680 |
|  | 3-oxoacid CoA-transferase subunit A, pcaI | CSV86_16050  CSV86_23554 |
|  | 3-oxoacid CoA-transferase subunit B, pcaJ | CSV86_23559 |
|  | beta-ketoadipyl CoA thiolase, pcaF | CSV86_16695 |
|  | benzoate transport | CSV86_16700 |
|  | Pca regulon regulatory protein PcaR | CSV86_16705 |
|  | 4-hydroxybenzoyl-CoA thioesterase domain-containing protein | CSV86_07451 |
|  | 5-carboxymethyl-2-hydroxymuconate isomerase | CSV86_13515 |
|  | Salicylate hydroxylase | CSV86_13595 |
| **Benzyl alcohol**  **pathway (119)** | Transcriptional regulator, AraC family | CSV86_16940 |
|  | Putative benzaldehyde dehydrogenase oxidoreductase protein | CSV86_16935 |
|  | AreB (aryl-alcohol dehydrogenase) | CSV86_16930 |
| **Phenylacetic acid**  **pathway**  **(88)** | Phenylacetic acid degradation operon negative regulatory protein PaaX | CSV86_11055 |
|  | Phenylacetic acid degradation protein PaaY | CSV86_11050 |
|  | enoyl-CoA hydratase isomerase | CSV86_11045 |
|  | enoyl-CoA hydratase | CSV86_11040 |
|  | 3-hydroxyacyl-CoA dehydrogenase PaaC | CSV86_11035 |
|  | Phenylacetic acid degradation protein PaaD | CSV86_11030 |
|  | beta-ketoadipyl CoA thiolase | CSV86_11025 |
|  | Phenylacetate-coenzyme A ligase, PaaF | CSV86_11020 |
|  | phenylacetate-CoA oxygenase subunit PaaA | CSV86_11005 |
|  | phenylacetate-CoA oxygenase subunit PaaB | CSV86_11000 |
|  | phenylacetate-CoA oxygenase, PaaI subunit | CSV86_10995 |
|  | phenylacetate-CoA oxygenase subunit PaaJ | CSV86_10990 |
|  | phenylacetate-CoA oxygenase/reductase, PaaK subunit | CSV86_10985 |
|  | acetate permease | CSV86_10975 |
|  | Phenylacetic acid-specific porin PaaM | CSV86_10970 |
|  | bifunctional aldehyde dehydrogenase/enoyl-CoA hydratase | CSV86_10965 |
| **4-Hydroxy**  **phenylacetate**  **(Homoproto-catechuate)**  **pathway**  **(7, 120, 175, 216)** | 4-hydroxyphenylacetate 3-monooxygenase operon regulatory protein, XylS/AraC family, hpaA | CSV86_00601 |
|  | 3,4-dihydroxyphenylacetate 2,3-dioxygenase, hpaD | CSV86_00621  CSV86_17587 |
|  | putative 4-hydroxyphenylacetate-3-monooxygenase protein | CSV86_23439 |
|  | putative permease transmembrane protein | CSV86_00631 |
|  | putative homoprotocatechuate degradative operon repressor | CSV86_00646 |
|  | homoprotocatechuate degradative operon repressor | CSV86_27878 |
|  | 2-oxo-hept-3-ene-1,7-dioate hydratase, hpaH | CSV86_00636 |
|  | 2,4-dihydroxyhept-2-ene-1,7-dioic acid aldolase, hpaI | CSV86_00641 |
|  | 5-carboxymethyl-2-hydroxymuconate delta-isomerase, hpcF | CSV86_00626 |
|  | 5-carboxymethyl-2-hydroxymuconate semialdehyde dehydrogenase, hpaE | CSV86_00616 |
|  | putative 5-carboxymethyl-2-oxo-hex-3-ene-1,7-dioate decarboxylase, hpaG | CSV86_00611 |
| **4-hydroxy benzoate**  **(Protocatechuate)**  **pathway**  **(99, 107, 118, 175)** | transcriptional regulator PobR | CSV86_14801 |
|  | 4-hydroxybenzoate 3-monooxygenase, pobA | CSV86_14796 |
|  | dicarboxylic acid transport protein, pcaT | CSV86_16690 |
|  | protocatechuate 3,4-dioxygenase, alpha subunit, pcaG | CSV86_12150 |
|  | protocatechuate 3,4-dioxygenase, beta subunit, pcaH | CSV86_12145 |
|  | 3-carboxy-cis,cis-muconate cycloisomerase, pcaB | CSV86_16685 |
|  | 4-carboxymuconolactone decarboxylase, pcaC | CSV86_16675 |
|  | 3-oxoadipate enol-lactonase, pcaD | CSV86_16680 |
|  | 3-oxoacid CoA-transferase subunit A, pcaI | CSV86_23554  CSV86_16050 |
|  | 3-oxoacid CoA-transferase subunit B, pcaJ | CSV86_23559 |
|  | beta-ketoadipyl CoA thiolase, pcaF | CSV86_16695 |
| **Phenyl propanoid**  **pathway**  **(115, 119)** | MarR family transcriptional regulator | CSV86_15610 |
|  | p-hydroxycinnamoyl CoA hydratase/lyase, ech | CSV86_15615 |
|  | Feruloyl-CoA synthetase, fcs | CSV86_15625 |
|  | Vanillin dehydrogenase, vdh | CSV86_15620 |
|  | vanillate monooxygenase, van AB | CSV86_16965 |
|  | vanillate O-demethylase oxygenase subunit, vanA | CSV86_28363 |
|  | vanillate O-demethylase oxidoreductase, vanB | CSV86_28368 |
|  | 5,10-methylenetetrahydrofolate reductase, metF | CSV86_12905 |
|  | GntR family transcriptional regulator | CSV86_28358 |
| **Homogentisate**  **pathway**  **(27, 99, 177,134)** | Homogentisate 1,2-dioxygenase, hmgA | CSV86_03097 |
|  | Fumarylacetoacetase, hmgB | CSV86_03102 |
|  | Maleylacetoacetate isomerase, hmgC | CSV86_03107 |
|  | Phenylalanine-4-hydroxylase, phhA | CSV86_23594 |
|  | transcriptional regulator TyrR | CSV86_23589 |
|  | pterin-4-alpha-carbinolamine dehydratase, phhB | CSV86_23599 |
|  | aromatic amino acid aminotransferase, phhC | CSV86_23604 |
|  | 4-hydroxyphenylpyruvate dioxygenase, hpd | CSV86_12190  CSV86_19308 |
