# Supplementary material for: Pseudomonas putida CSV86: A Candidate Genome for Genetic Bioaugmentation
Source: PLoS One. 2014 Jan 24;9(1):e84000. doi: 10.1371/journal.pone.0084000 (PMC3901652; doi:10.1371/journal.pone.0084000)
Supplement: Table S3 — Percentage homology of Naphthalene upper and lower operon pathway proteins in P. putida CSV86 with homologous proteins of 4 Pseudomonas species reported for naphthalene degradation capability. (DOCX) [file pone.0084000.s019.docx]

**Table S3**. Percentage homology of Naphthalene upper and lower operon pathway proteins in *P. putida* CSV86 with homologous proteins of 4 *Pseudomonas* species reported for naphthalene degradation capability.

**A. upper pathway proteins (*nah* operon)**

| **Sl. No** | **Proteins**  **Upper Pathway** | ***P. aeruginosa* D84146**  **(%)** | ***P.stutzeri* AF039533**  **(%)** | ***P. fluorscens* strain PC20 plasmid p Nah20**  **AY887963**  **(%)** | ***P. putida* plasmid NAH7 DNA strain G7**  **AB237655**  **(%)** |
| --- | --- | --- | --- | --- | --- |
| 1. | NahAa | 100 | 99 | 86 | 86 |
| 2. | NahAb | 100 | 100 | 90 | 92 |
| 3. | NahAc | 99 | 98 | 90 | 90 |
| 4. | NahAd | 99 | 98 | 82 | 80 |
| 5. | NahB | 98 | 98 | 91 | 92 |
| 6. | NahF | 97 | 98 | 92 | 93 |
| 7. | NahC | 91 | 98 | 91 | 91 |
| 8. | NahE | 93 | 99 | 93 | 94 |
| 9. | NahD | 81 | 100 | 81 | 78 |

**B. lower pathway proteins (*sal* operon)**

| **Sl. No** | **Proteins**  **Lower Pathway** | ***P. aeruginosa* strain CGMCC 1.860 plasmid salicylate GQ396161**  **(%)** | ***P.stutzeri* transposase like protein**  **AF039534**  **(%)** | ***P. fluorscens* strain PC20 plasmid p Nah20**  **AY887963**  **(%)** | ***P. putida* plasmid NAH7 DNA strain G7**  **AB237655**  **(%)** |
| --- | --- | --- | --- | --- | --- |
| 1. | NahR | 100 | 100 | 84 | 81 |
| 2. | NahG | 99 | 99 | 82 | 84 |
| 3. | NahJ | 100 | 100 | 84 | 84 |
| 4. | Cat2,3 | 99 | 99 | 89 | 88 |
| 5. | NahI | 99 | 99 | 96 | 93 |
| 6. | NahN | 97 | 97 | 79 | 79 |
| 7. | NahL | 97 | 97 | 88 | 88 |
| 8. | NahM | 99 | 99 | 94 | 95 |
| 9. | NahO | 99 | 100 | 96 | 96 |
| 10. | NahK | 97 | 97 | 94 | 95 |
| 11. | NahJ | - | 95 | 75 | 78 |
