# Supplementary material for: Pseudomonas putida CSV86: A Candidate Genome for Genetic Bioaugmentation
Source: PLoS One. 2014 Jan 24;9(1):e84000. doi: 10.1371/journal.pone.0084000 (PMC3901652; doi:10.1371/journal.pone.0084000)
Supplement: Table S4 — Heavy metal resistance genes identified in P. putida CSV86 genome annotation in RAST and their percentage homology with the closest respective gene. (DOCX) [file pone.0084000.s020.docx]

**Table S4.** Heavy metal resistance genes identified in *P. putida* CSV86 genome annotation in RAST and their percentage homology with the closest respective gene.

| **Heavy metal resistance Genes** | **Start** | **End** | **Size**  **(nt)** | **Contig** | **% Match**  **With closest respective gene in RAST** |
| --- | --- | --- | --- | --- | --- |
| Arsenic resistance protein ArsH | 1540 | 839 | 702 | 00153 | *P. putida* KT2440  611/700 (87%) |
| Arsenic efflux pump protein | 3334 | 2051 | 1284 | 00153 | *P. putida* F1  1114/1286 (87%) |
| Arsenical resistance operon repressor | 3703 | 3356 | 348 | 00153 | *P. putida* KT2440  290/348 (83%) |
| Arsenate reductase | 2023 | 1553 | 471 | 00153 | *P. putida* F1  399/471 (85%) |
| Arsenate reductase | 14215 | 14568 | 354 | 00082 | *P. putida* GB-1  300/355 (85%) |
| Copper sensory histidine kinase CusS | 7729 | 6389 | 1341 | 00019 | *P. putida* W619  972/1301 (75%) |
| Copper-sensing two-component system response regulator CusR | 8409 | 7729 | 681 | 00019 | *P. putida* GB-1  563/671 (84%) |
| Copper tolerance protein | 8560 | 9045 | 486 | 00019 | *P. putida* W619  340/485 (70%) |
| Copper-sensing two-component system response regulator CusR | 28057 | 27380 | 678 | 00019 | *P. putida* KT2440  524/678 (77%) |
| Copper tolerance protein | 28792 | 29349 | 558 | 00019 | *P. mendocina* ymp  552/558 (99%) |
| Copper tolerance protein | 29497 | 30030 | 534 | 00019 | *P. mendocina* ymp  530/534 (99%) |
| Lead, cadmium, zinc and mercury transporting ATPase | 30643 | 32724 | 2082 | 00019 | *P. mendocina* ymp  1975/2082 (95%) |
| Multicopper oxidase | 39347 | 41161 | 1815 | 00019 | *P. mendocina* ymp  1671/1815 (92%) |
| Copper resistance protein B | 41442 | 42566 | 1125 | 00019 | *P. mendocina* ymp  1091/1125 (97%) |
| Copper homeostasis protein CutE | 59644 | 61161 | 1518 | 00022 | *P. fluorescens* Pf-5  1243/1517 (82%) |
| Lead, cadmium, zinc and mercury transporting ATPase | 20226 | 18229 | 1998 | 00024 | *P. putida* KT2440  1596/1998 (80%) |
| Lead, cadmium, zinc and mercury transporting ATPase | 8276 | 6279 | 1998 | 00043 | *P. putida* KT2440  1973/1998 (99%) |
| Heavy-metal-associated domain (N-terminus) and membrane-bounded cytochrome biogenesis cycZ-like domain, possible membrane copper tolerance protein | 43536 | 42853 | 684 | 00049 | *P. putida* W619  573/679 (84%) |
| Copper-translocating P-type ATPase | 46207 | 43754 | 2454 | 00049 | *P. fluorescens* PfO-1  1982/2404 (82%) |
| Lead, cadmium, zinc and mercury transporting ATPase | 16473 | 18599 | 2127 | 00060 | *P. putida* F1  1714/2136 (80%) |
| Lead, cadmium, zinc and mercury transporting ATPase | 24195 | 21991 | 2205 | 00114 | *P. entomophila* L48  1769/2168 (82%) |
| Multicopper oxidase | 75054 | 76757 | 1704 | 00116 | *P. putida* GB-1  1370/1700 (81%) |
| copper resistance protein B precursor | 76774 | 77688 | 915 | 00116 | *P. entomophila* L48  635/808 (79%) |
| Lead, cadmium, zinc and mercury transporting ATPase | 2661 | 712 | 1950 | 00131 | *P. entomophila* L48  1541/1929 (80%) |
| Multicopper oxidase | 12096 | 13478 | 1383 | 00154 | *P. putida* W619  1200/1386 (87%) |
| Chromate transport protein ChrA | 12080 | 10716 | 1365 | 00005 | *P. mendocina* ymp  1019/1303 (78%) |
| Chromate transport protein ChrA | 29772 | 28561 | 1212 | 00186 | *P. mendocina* ymp  914/1180 (77%) |
| Cobalt-zinc-cadmium resistance protein CzcA; Cation efflux system protein CusA | 49139 | 45990 | 3150 | 00019 | *P. mendocina* ymp  2857/3150 (91%) |
| Cobalt/zinc/cadmium efflux RND transporter, membrane fusion protein, CzcB family | 50596 | 49136 | 1461 | 00019 | *P. mendocina* ymp  1225/1462 (84%) |
| Cobalt-zinc-cadmium resistance protein | 44180 | 45052 | 873 | 00022 | *P. entomophila* L48  656/857 (77%) |
| Cobalt-zinc-cadmium resistance protein CzcD | 7286 | 8191 | 906 | 00024 | *P. putida* CSV86  749/906 (83%) |
| Cobalt-zinc-cadmium resistance protein CzcA; Cation efflux system protein CusA | 24066 | 20905 | 3162 | 00024 | *P. putida* KT2440  2789/3155 (88%) |
| Cobalt/zinc/cadmium efflux RND transporter, membrane fusion protein, CzcB family | 25341 | 24091 | 1251 | 00024 | *P. putida* CSV86  1071/1251 (86%) |
| Cobalt-zinc-cadmium resistance protein CzcD | 93835 | 94740 | 906 | 00036 | *P. putida* W619  632/892 (71%) |
| Cobalt-zinc-cadmium resistance protein CzcD | 6924 | 6019 | 906 | 00042 | *P. putida* KT2440  899/906 (99%) |
| Cobalt-zinc-cadmium resistance protein CzcA; Cation efflux system protein CusA | 12125 | 8964 | 3162 | 00043 | *P. putida* KT2440  3093/3162 (98%) |
| Cobalt/zinc/cadmium efflux RND transporter, membrane fusion protein, CzcB family | 13400 | 12150 | 1251 | 00043 | *P. putida* KT2440  1197/1251 (96%) |
| Cobalt-zinc-cadmium resistance protein CzcD | 994 | 461 | 534 | 00112 | *Acidovorax sp.* JS42  275/404 (68%) |
| Cobalt-zinc-cadmium resistance protein | 43978 | 44928 | 951 | 00118 | *P. putida* KT2440  740/912 (81%) |
| Cobalt-zinc-cadmium resistance protein CzcD | 1437 | 682 | 756 | 00155 | *P. putida* W619  756/756 (100%) |
| Cobalt-zinc-cadmium resistance protein CzcA; Cation efflux system protein CusA | 34883 | 37999 | 3117 | 00172 | *P. putida* W619  2553/3039 (84%) |
| Cobalt-zinc-cadmium resistance protein CzcA; Cation efflux system protein CusA | 29443 | 26294 | 3150 | 00220 | *P. putida* KT2440  2498/3121 (80%) |
| Cobalt/zinc/cadmium efflux RND transporter, membrane fusion protein, CzcB family | 30679 | 29468 | 1212 | 00220 | *P. putida* GB-1  906/1252 (72%) |
| Chromate transport protein ChrA | 12080 | 10716 | 1365 | 00005 | *P. putida* GB-1  1035/1296(80%) |
| Chromate transport protein ChrA | 12080 | 10716 | 1365 | 00186 | *P. mendocina* ymp  914/1180(77%) |
