# Supplementary material for: Pseudomonas putida CSV86: A Candidate Genome for Genetic Bioaugmentation
Source: PLoS One. 2014 Jan 24;9(1):e84000. doi: 10.1371/journal.pone.0084000 (PMC3901652; doi:10.1371/journal.pone.0084000)
Supplement: Table S5 — Mobile genetic elements present in P. putida CSV86 genome located using IS finder. (DOCX) [file pone.0084000.s021.docx]

**Table S5.** Mobile genetic elements present in *P. putida* CSV86 genome located using IS finder.

| **Family** | **Name** | **Length** | **Presence in CSV86** | **Reference/origin** | **Properties associated** |
| --- | --- | --- | --- | --- | --- |
| Tn*3* | IS*Pa42* | 16941 | Contig 19 | *Pseudomonas aeruginosa* | Not reported |
| IS*66* | IS*Pre3* | 2957 | Contig 232 | *Pseudomonas resinovorans* | Found in carbazole-degradative plasmid pCAR1 in *Pseudomonas resinovorans* (1) |
| IS*L3* | IS*Ppu12* | 3372 | Contig 155 | *Pseudomonas putida* | Found in the toluene-xylene catabolic plasmid pWW0 of *Pseudomonas putida* mt-2 (2) |
| IS*L3* | IS*Pst2* | 2984 | Contig 242 | *Pseudomonas stutzeri* M1 | It inactivates the *m*-xylene and *p*-xylene catabolic pathway as well as the *o*-xylene catabolic genes in its derivative strain M1 (3) |
| IS*L3* | IS*1396* | 1771 | Contig 187 | *Serratiamarcescens* (pR471a) | IS*1396* is widely distributed and has been found in genomes of gram-positive bacteria, *Cyanobacteria*, and broadhost-range plasmids from gram-negative bacteria. It may be involved in assembly of the *ortho*-chlorobenzoate pathway. (4) |
| IS*L3* | IS*Pst9* | 2472 | Contig 187 | *Pseudomonas stutzeri* | IS*Pst*9 has been shown to be responsible for the *nahH* insertional inactivation in 4-chlorosalicylate-degrading derivative strain AN142 (5) |
| IS*5* | IS*Ach1* | 1192 | Contig 161 | *Achromobacter sp.* | Not reported |
| IS*3* | [IS*Pa20*](http://www-is.biotoul.fr/is/scripts/is_spec.php?name=ISPa20) | 1246 | Contig 19 | *Pseudomonas aeruginosa* | Isolated from *P.aeruginosa* clone C subclone C13 strains in cystic fibrosis patients. It was shown to be involved in genomic reorganization and horizontal gene transfer (6) |
| Tn*3* | IS*Pa40* | 6592 | Contig 101 | *Pseudomonas aeruginosa* | Not reported |

**References:**

1. Shintani M, Matsumoto T, Yoshikawa H, Yamane H, Ohkuma M (2011) DNA rearrangement has occurred in the carbazole-degradative plasmid pCAR1and the

chromosome of its unsuitable host, *Pseudomonas fluorescens* Pf0-1. Microbiology 157: 3405-3416.

1. Williams PA, Jones RM, Shaw LE (2002) A third transposable element, IS*Ppu12*, from the toluene-xylene catabolic plasmid pWW0 of *Pseudomonas putida* mt-2. J Bacteriol 184: 6572-

6580.

1. Bolognese F, Di Lecce C, Galli E, Barbieri P (1999) Activation and inactivation of *Pseudomonas stutzeri* methylbenzene catabolism pathways mediated by a transposable element. Appl Environ Microbiol 65: 1876-1882.
2. Tsoi TV, Plotnikova EG, Cole JR, Guerin WF, Bagdasarian M (1999) Cloning, expression, and nucleotide sequence of the *Pseudomonas aeruginosa*142 *ohb* genes

coding for oxygenolytic *ortho* dehalogenation of halobenzoates. Appl Environ Microbiol 65: 2151-2162.

1. Christie-Oleza JA, Nogales B, Martín-Cardona C, Lanfranconi MP, Albertí S, et al. (2008) IS*Pst9*, an IS*L3* like insertion sequence from *Pseudomonas stutzeri* AN10 involved in catabolic gene inactivation. Int Microbiol 11: 101-110.
2. Kresse AU, Blöcker H, Römling U (2006) IS*Pa20* advances the individual evolution of *Pseudomonas aeruginosa* clone C subclone C13 strains isolated from cystic fibrosis patients by insertional mutagenesis and genomic rearrangements. Arch Microbiol 185: 245-254.
